# Artificial intelligence–assisted smart hydrogel bioinks in 3D bioprinting: design, optimization, and construct validation for functional tissue engineering

**DOI:** 10.3389/fbioe.2026.1898243

**Published:** 2026-08-10

**Authors:** Siyuan Zhang, Dingwen Liang, Yingying Lei, Yuxi Luo, Rui Shi

**Affiliations:** 1 Department of IT & Data Management, West China Hospital, Sichuan University, Chengdu, China; 2 Information Center, Beijing Anzhen Nanchong Hospital, Capital Medical University & Nanchong Central Hospital, Nanchong, China; 3 Department of Neurology, The Second Clinical Medical College, Ningxia Medical University, Yinchuan, China; 4 Department of Medical Oncology, National Cancer Center/National Clinical Research Center for Cancer/Cancer Hospital, Chinese Academy of Medical Sciences and Peking Union Medical College, Beijing, China

**Keywords:** 3D bioprinting, artificial intelligence, bioinks, smart hydrogels, tissue engineering

## Abstract

Hydrogel-based bioinks are central to three-dimensional (3D) bioprinting because they provide hydrated, cell-supportive microenvironments with tunable rheological, mechanical, and biological properties. Smart hydrogel bioinks further introduce stimuli-responsive and dynamic behaviors, but their development remains constrained by empirical trial-and-error workflows and weak integration among formulation design, printability, process monitoring, and post-print biological performance. This review develops an AI-assisted workflow framework for smart hydrogel bioink development rather than treating smart materials, algorithms, and autonomous laboratories as separate mature topics. We examine how artificial intelligence (AI) can support feature representation, property prediction, printability assessment, process optimization, monitoring, construct characterization, and iterative refinement. Particular emphasis is placed on distinguishing direct evidence in smart hydrogel bioinks from broader bioprinting evidence, adjacent-field methodological inspiration, and prospective autonomous concepts. We also clarify the boundaries among supervised prediction, Bayesian optimization, active learning, computer vision, feedback control, and AI-agent-assisted workflow coordination. Finally, we discuss validation, benchmarking, grouped data splitting, uncertainty estimation, out-of-distribution detection, and the need to connect early material and process descriptors with long-term biological function. Overall, AI-assisted methods can make hydrogel bioprinting more predictive and quality-oriented, but real-time closed-loop control and fully autonomous bioink laboratories remain prospective goals that require standardized datasets, validated biological endpoints, uncertainty-aware models, external validation, and human oversight.

## 1 Introduction

Three-dimensional (3D) bioprinting is an important biofabrication strategy for tissue engineering and related biomedical applications because it enables the spatially controlled deposition of cells, biomaterials, and bioactive cues into predefined architectures (Mandrycky et al., 2016; Zhang et al., 2017). Compared with conventional scaffold fabrication approaches, it offers better control of construct geometry, compositional heterogeneity, and cell distribution, enabling researchers to engineer tissue-like microenvironments with improved structural and biological relevance (Bishop et al., 2017). As the field moves toward more reproducible and application-oriented platforms, bioink properties and design logic have become central to both printing success and downstream construct performance (Gungor-Ozkerim et al., 2018; Unagolla and Jayasuriya, 2020; Hull et al., 2022).

Hydrogels remain the most widely used bioinks because of their high water content, extracellular matrix-like features, favorable cytocompatibility, and broad tunability in rheological, mechanical, and biochemical properties (Fang et al., 2023). These features make hydrogel-based bioinks attractive for regenerative tissue constructs, *in vitro* disease models, drug screening, and personalized testing (Ma et al., 2018; Satpathy et al., 2018; Lam et al., 2023; Sztankovics et al., 2023). However, hydrogel bioink development still relies heavily on empirical trial-and-error optimization, and material selection, formulation adjustment, printing parameter tuning, and biological evaluation are often conducted separately (Bian, 2020; Hull et al., 2022; Freeman et al., 2022). This fragmented workflow is slow, difficult to scale, and poorly suited to resolve trade-offs among printability, structural fidelity, cell compatibility, and tissue-specific function (Freeman et al., 2022; Fang et al., 2023). To address these limitations, increasing attention has been directed toward smart hydrogel bioinks, which extend beyond passive structural support through programmable, adaptive, or multifunctional behavior (Malekmohammadi et al., 2021; Bian, 2020; Mohamed et al., 2019). Depending on their design, smart hydrogels can exhibit stimuli responsiveness, dynamic or reversible crosslinking, self-healing capacity, tunable mechanics, controlled factor release, or improved regulation of cell behavior post-printing (Malekmohammadi et al., 2021; Bian, 2020; Mohamed et al., 2019). These features are relevant to 3D bioprinting, where performance depends not only on intrinsic material properties but also on their interactions with processing conditions and biological outcomes. However, this broader design space increases complexity, because polymer composition, concentration, crosslinking strategy, rheological behavior, and bioactivity are often strongly interdependent (Hull et al., 2022; Fang et al., 2023).

Artificial intelligence (AI) is increasingly being explored to accelerate and refine hydrogel bioink development (Freeman et al., 2022; Yu and Jiang, 2020; An et al., 2021; Sun et al., 2023). AI-assisted approaches can integrate material descriptors, formulation parameters, rheological measurements, imaging outputs, process variables, and biological readouts to support property prediction, formulation screening, parameter optimization, process monitoring, defect detection, construct characterization, and functional evaluation (Chen et al., 2023; Ning et al., 2023). These applications should not be treated as equivalent levels of automation. Most current studies support one or a few modules, such as prediction, offline optimization, image analysis, or monitoring, whereas systems that combine real-time sensing, automated state estimation, online parameter adjustment, and repeated feedback during the same fabrication process remain comparatively limited. For smart hydrogel bioinks, closed-loop strategies may eventually improve process control, but the stronger vision of autonomous bioink laboratories should be framed as an emerging and prospective objective rather than a mature capability. Despite these opportunities, AI-assisted hydrogel bioprinting remains constrained by small and heterogeneous datasets, inconsistent reporting standards, limited cross-platform generalizability, insufficient integration of multimodal data, poor model interpretability, and unresolved translational and regulatory challenges (Freeman et al., 2022; Chen et al., 2023). In addition, better printability or process control does not necessarily translate into meaningful tissue-level outcomes, underscoring the need for stronger construct characterization and function-oriented validation frameworks (Hull et al., 2022; Fang et al., 2023).

**FIGURE 1:**
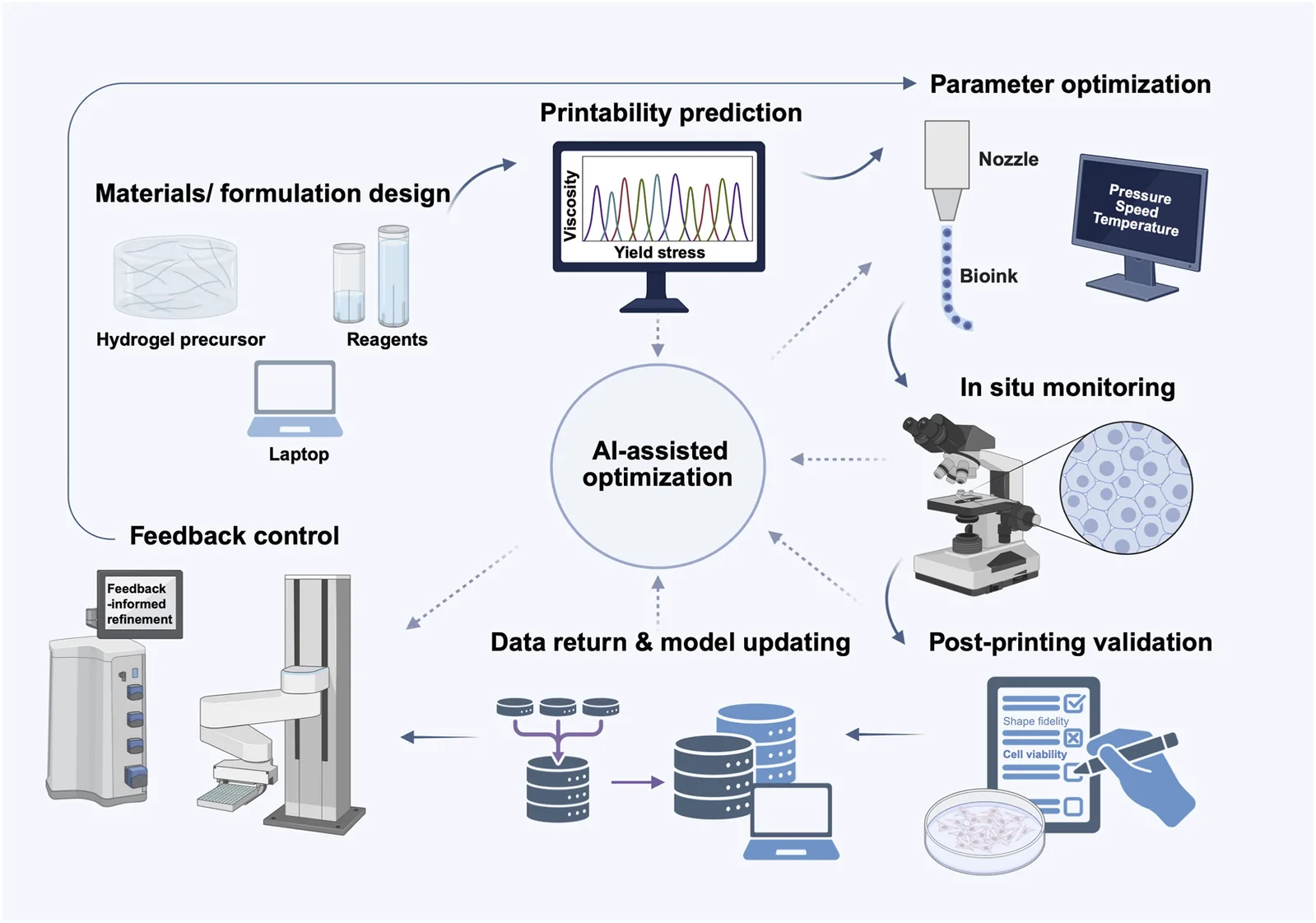
AI-assisted workflow for smart hydrogel bioink design, bioprinting, monitoring, and feedback-informed refinement. The schematic shows how AI can support materials/formulation design, property and printability prediction, process optimization, in situ monitoring, post-printing validation, and data return for between-run model updating. Post-printing data feedback should be interpreted as feedback-informed refinement unless it is coupled to automated in-process sensing, decision-making, and online adjustment of printing parameters or tool paths. Created in BioRender. Luo, Y. (2026) https://BioRender.com/bhk6ddb.

To avoid ambiguity, this review uses the terms closed-loop and autonomous cautiously. Strict feedback control is defined as real-time sensing, automated analysis or state estimation, automated decision-making, online adjustment of printing parameters or tool paths, and repeated in-process feedback within the same fabrication run. Post-printing results that guide later experiments are instead described as between-run iterative learning, feedback-informed refinement, or iterative model updating. Based on this distinction, the review develops a workflow framework that connects formulation design, feature representation, property prediction, printability assessment, process optimization, monitoring, construct validation, uncertainty, generalizability, and translational challenges. Figure 1 summarizes the AI-assisted workflow framework used in this review, from materials and formulation design to printability prediction, parameter optimization, *in situ* monitoring, post-printing validation, and feedback-informed model updating.

To reduce over-extrapolation, the review also applies an evidence hierarchy. Studies directly involving smart hydrogel bioinks or closely matched hydrogel-bioprinting systems are treated as direct evidence. Broader extrusion-bioprinting, printability, monitoring, or construct-assessment studies are considered broader bioprinting evidence. Work from materials informatics, drug discovery, robotic chemistry, general hydrogel science, and non-bioprinting imaging is treated as adjacent-field methodological inspiration. Fully autonomous bioink discovery, agent-driven laboratories, and general-purpose foundation-model deployment are therefore discussed as prospective concepts unless validated in smart hydrogel bioink-relevant workflows.

## 2 Smart hydrogels as bioinks: design logic and key performance requirements

Hydrogels remain one of the most widely used material platforms in 3D bioprinting because they provide a highly hydrated microenvironment, support cell encapsulation, and partially mimic the extracellular matrix of native tissues. Their broad utility arises from the ability to combine structural support with biological permissiveness, which can enable the fabrication of constructs that sustain cell survival, proliferation, and differentiation. As a result, hydrogels are widely regarded as a cornerstone of bioink development, although their successful application depends critically on balancing printability, mechanical stability, and biological performance (Unagolla and Jayasuriya, 2020; Schwab et al., 2020; Gogoi et al., 2024). Within this broader class, smart hydrogels, also known as stimuli-responsive hydrogels, have attracted increasing attention for their ability to dynamically modulate physicochemical properties in response to internal or external cues. These materials respond to temperature, pH, light, enzymatic activity, redox conditions, ionic strength, and other environmental signals, enabling adaptive regulation of gelation, swelling, degradation, stiffness, permeability, and bioactive factor release. More advanced systems exhibit additional functionalities, including self-healing, dynamic crosslinking, shape-memory, and environment-adaptive remodeling, which allow them to adapt to evolving biological and manufacturing conditions (Neumann et al., 2023; Wu et al., 2024).

**FIGURE 2:**
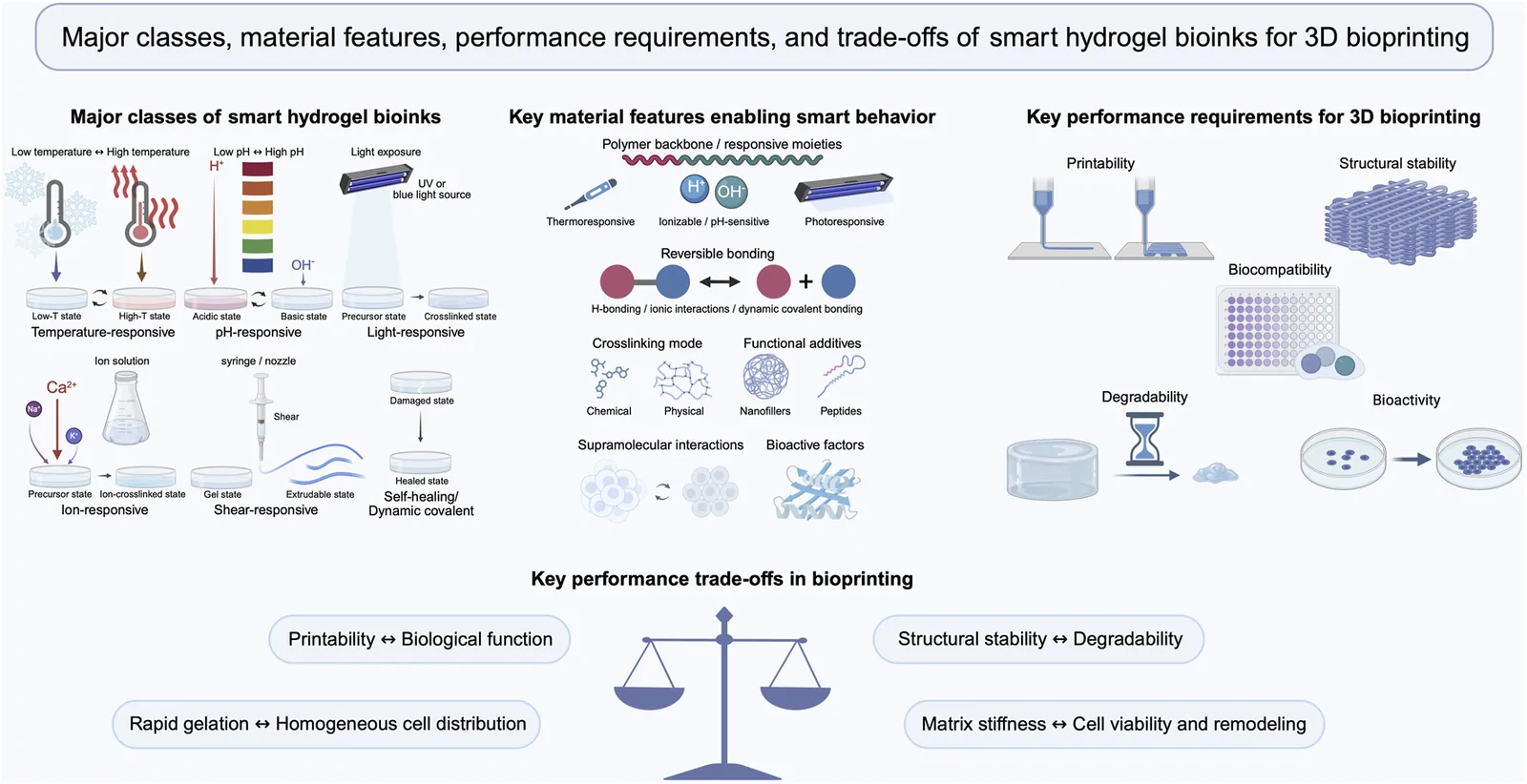
Major classes, material features, performance requirements, and trade-offs of smart hydrogel bioinks for 3D bioprinting. The schematic summarizes representative smart hydrogel bioinks, key material features, major bioprinting requirements, and design trade-offs. It highlights responsive and dynamic hydrogel systems, including temperature-, pH-, light-, ion-, and shear-responsive bioinks, as well as self-healing and dynamic covalent systems. It also emphasizes trade-offs among printability, biological function, gelation, structural stability, degradability, matrix stiffness, and cell viability/remodeling, underscoring the complexity of smart hydrogel bioink design and the value of AI-assisted optimization. Created in BioRender. Luo, Y. (2026) https://BioRender.com/9bvdq6v.

These capabilities make smart hydrogels useful for functional tissue engineering, where the microenvironment is inherently dynamic and spatially heterogeneous. In addition to serving as structural scaffolds, smart hydrogel bioinks can act as programmable platforms for controlled delivery, matrix remodeling, and post-printing functional regulation (Neumann et al., 2023; Gogoi et al., 2024; Wu et al., 2024). These advantages also introduce design complexity. Smart hydrogel bioinks are governed by many interdependent variables, including material composition, crosslinking chemistry, processing conditions, and biological components. The relationships among formulation parameters, rheological behavior, printability, and biological performance are highly nonlinear and often involve competing objectives, such as balancing structural fidelity with cell viability or mechanical strength with matrix remodeling (Gogoi et al., 2024; Schwab et al., 2020). Moreover, their spatiotemporal responsiveness means that material properties may evolve during and after the printing process, further complicating prediction and optimization (Neumann et al., 2023). As summarized in Figure 2, the design of smart hydrogel bioinks is governed by the interplay among responsive material classes, enabling structural features, bioprinting-related performance requirements, and multiple trade-offs between competing objectives. To further clarify the concept of smart hydrogels, we next discuss their definition and distinguishing features in more detail.

### 2.1 Definition and distinguishing features of smart hydrogel bioinks

A smart hydrogel bioink should be distinguished from a smart hydrogel more generally. In this review, the term smart hydrogel bioink refers to a hydrogel formulation that simultaneously satisfies three conditions: it has printability suitable for 3D bioprinting, it can carry or support cells and relevant biological components, and it provides controllable, dynamic, or stimulus-responsive functions. These functions may include reversible or dynamic crosslinking, self-healing, programmable degradation, controlled release, adaptive mechanical remodeling, or responses to temperature, pH, light, ionic concentration, enzymatic activity, redox state, or other environmental cues (Koetting et al., 2015; El-Husseiny et al., 2022).

This distinction is important because a smart hydrogel is not automatically a smart hydrogel bioink. A hydrogel may show stimulus responsiveness yet still fail as a bioink if it lacks printable rheology, cytocompatibility, homogeneous cell encapsulation, stable deposition, or reproducible manufacturing behavior (Schwab et al., 2020; Hölzl et al., 2016). Conventional bioinks mainly provide relatively passive structural and biological support during fabrication and culture. By contrast, smart hydrogel bioinks add time-varying, tunable, or environment-responsive functions that can regulate gelation, recovery, degradation, permeability, factor release, or cell-matrix interactions during or after printing. Responsiveness alone is therefore insufficient; the responsive function must be compatible with the manufacturing and biological requirements of the intended bioprinting application.

### 2.2 Major classes of smart hydrogel bioinks

Smart hydrogel bioinks can be classified along two orthogonal dimensions: smart functionality and material origin. Classification by smart functionality focuses on the response mechanism or dynamic behavior, such as thermo-responsiveness, photo-responsiveness, pH-responsiveness, ion-responsiveness, enzyme- or redox-responsiveness, self-healing, shear responsiveness, or dynamic covalent exchange (Koetting et al., 2015; Neumann et al., 2023). Classification by material origin focuses on whether the network is naturally derived, synthetic, or hybrid/composite (Groll et al., 2016; Schwab et al., 2020). These dimensions are non-exclusive. For example, one formulation may be naturally derived, photo-responsive, self-healing, and dynamically crosslinked at the same time.

Classification by smart functionality is useful because the trigger or mechanism determines how the bioink behaves during extrusion, stabilization, culture, and remodeling. Thermo-responsive systems can regulate sol-gel transitions; photo-responsive systems, including gelatin methacryloyl (GelMA)-based photocrosslinkable hydrogels, enable spatial and temporal control of network formation; pH-, enzyme-, redox-, and ion-responsive systems can support biochemical adaptation, controlled degradation, release, or rapid stabilization; and shear-responsive, self-healing, or dynamic covalent systems can improve extrusion and post-print recovery (Koetting et al., 2015; Yue et al., 2015; Liu et al., 2023). Each functionality also carries limitations, such as sensitivity to process temperature, phototoxicity, heterogeneous gelation, variable biological triggers, or tension between rapid recovery and long-term mechanical stability.

Classification by material origin captures a different design axis. Natural-derived polymers such as alginate, hyaluronic acid, collagen, gelatin derivatives, and agarose provide biomimetic and cell-supportive environments but may show batch variability or weaker control over mechanics. Synthetic systems provide more tunable chemistry, mechanics, and degradation kinetics but may require additional modification to support cell adhesion and bioactivity. Hybrid or composite systems combine these advantages and can incorporate particles, nanomaterials, dynamic linkages, or multiple crosslinking mechanisms, but they also increase formulation complexity and validation burden (Groll et al., 2016; Schwab et al., 2020). Table 1 therefore separates functional and material-origin classifications while emphasizing that an individual bioink can belong to multiple categories simultaneously.

**TABLE 1:** Classification of smart hydrogel bioinks by smart functionality and material origin.

Panel	Representative systems	Trigger or mechanism	Bioprinting relevance or main strengths	Main limitations	Representative references
Panel A. Classification by smart functionality					
Thermo-responsive	Gelatin-based and PNIPAAm-related systems	Temperature-induced sol-gel transition	Mild processing and convenient gelation control	Temperature sensitivity may affect print fidelity and cell compatibility	Koetting et al. (2015), Schwab et al. (2020)
Photo-responsive	GelMA and related photocrosslinkable hydrogels	Light-triggered crosslinking	Spatiotemporal control and rapid stabilization	Photoinitiator or light exposure may affect cells	Koetting et al. (2015), Yue et al. (2015)
pH-responsive	pH-sensitive polymer networks	pH-dependent swelling, gelation, or degradation	Adaptive release and local microenvironment response	Often limited in printing robustness	Koetting et al. (2015), El-Husseiny et al. (2022)
Ion-responsive	Alginate-related and ionically crosslinked hydrogels	Ionic crosslinking	Fast gelation and simple processing	Rapid gelation may compromise homogeneous cell distribution	Groll et al. (2016), Hölzl et al. (2016)
Enzyme-responsive	Biochemically labile hydrogels	Enzyme-mediated cleavage or remodeling	Biologically triggered degradation or release	Trigger levels vary across biological systems	Koetting et al. (2015), Liu et al. (2023)
Redox-responsive	Redox-sensitive hydrogel networks	Redox-triggered remodeling	Potentially useful for pathological or oxidative microenvironments	Requires careful biological validation	Koetting et al. (2015), Liu et al. (2023)
Self-healing or shear-responsive	Supramolecular and physically dynamic hydrogels	Shear thinning and post-shear recovery	Improved extrudability and post-print recovery	Recovery and long-term stability must be balanced	Leijten et al. (2017), Neumann et al. (2023)
Dynamic covalent systems	Reversible covalent or hybrid adaptive hydrogels	Bond exchange and adaptive remodeling	Tunable mechanics and post-print adaptability	Greater formulation complexity	Liu et al. (2023), Wu et al. (2024)
Panel B. Classification by material origin					
Natural-derived	Alginate, hyaluronic acid, collagen, gelatin derivatives, agarose	Biomimetic polymer origin	Good biocompatibility and bioactivity	Batch variability and weaker mechanical control	Groll et al. (2016), Schwab et al. (2020)
Synthetic	Chemically defined polymer networks	Programmable network chemistry	Better control over mechanics, degradation, and crosslinking	May require biofunctionalization	Groll et al. (2016), Schwab et al. (2020)
Hybrid or composite systems	Natural-synthetic blends and reinforced networks	Combined matrix and engineered components	Balances biomimicry, tunability, and functionality	Higher formulation and validation burden	Groll et al. (2016), Schwab et al. (2020)

These classification dimensions are non-exclusive, and an individual formulation may belong to multiple functional and material-origin categories simultaneously. Abbreviations: GelMA, gelatin methacryloyl; PNIPAAm, poly (N-isopropylacrylamide).

### 2.3 Key performance requirements of hydrogel bioinks for 3D bioprinting

The design of hydrogel bioinks is inherently a multi-objective optimization problem, requiring simultaneous satisfaction of printability, structural fidelity, cell protection, and biological activity. These requirements are often difficult to balance. Increased viscosity can improve shape retention, but it can also increase extrusion pressure and cellular shear stress. Increased yield stress can improve structural stability after deposition, but may restrict cell migration and matrix remodeling. Rapid gelation can improve early shape retention, but may cause nozzle clogging, heterogeneous cell distribution, or premature crosslinking. High crosslinking density can improve mechanical stability, but may reduce nutrient transport, oxygen diffusion, cell spreading, and matrix remodeling. Increased stiffness can also limit proliferation, differentiation, or phenotypic maturation, depending on the intended tissue context (Theus et al., 2020; Schwab et al., 2020; Wang et al., 2023).

Rheological behavior remains one of the most important determinants of bioink performance. Parameters such as viscosity, shear-thinning behavior, yield stress, viscoelasticity, and recovery kinetics strongly influence extrusion stability, filament continuity, and post-deposition shape retention. Crosslinking behavior and post-printing stability are similarly critical because gelation must be compatible with both the printing process and embedded cells. Prolonged light exposure or higher photoinitiator concentration may increase phototoxicity and oxidative stress, while slow degradation may obstruct new matrix deposition and tissue remodeling (Billiet et al., 2014; Dubbin et al., 2017; Galarraga et al., 2019). Immediate post-print viability is necessary but insufficient to demonstrate biological success. Longer-term evaluation should include proliferation, apoptosis, phenotype maintenance, migration, differentiation, extracellular matrix deposition, tissue maturation, and tissue-specific function. Current bioink design therefore requires coordinated tuning of rheology, crosslinking dynamics, transport, degradation, and biological function, supporting integrative predictive approaches, including AI-assisted design.

## 3 AI for smart hydrogel bioink formulation design and property prediction

### 3.1 Data foundations and feature representation for AI-assisted bioink design

AI-assisted design of smart hydrogel bioinks depends on high-quality, structured, and diverse datasets. Unlike conventional materials, hydrogel bioinks are highly multivariable and context-dependent, with performance shaped not only by composition but also by polymer concentration, molecular architecture, crosslinking strategy, rheological behavior, printing conditions, and biological context. As a result, AI models must learn from heterogeneous data spanning formulation, processing, and functional outcomes, making the data foundation a major determinant of model reliability and generalizability (Wang et al., 2025; Ramesh et al., 2024).

Current data sources mainly include published literature, laboratory-generated datasets, and emerging multimodal datasets integrating physicochemical, rheological, printing, imaging, and biological readouts. Literature-derived datasets provide broader formulation coverage but are often limited by inconsistent reporting, incomplete process descriptions, and non-uniform endpoint definitions. By contrast, single-laboratory datasets are usually more internally consistent but remain smaller and less diverse. Feature representation is therefore a central challenge because predictive models must encode formulation composition, material behavior, printing parameters, and biological context as machine-readable inputs (Wang et al., 2025; Zhang et al., 2025a; Zhang et al., 2025b). Static or single-point descriptors may include polymer identity, polymer concentration, molecular weight, degree of substitution, initial viscosity, yield stress, storage and loss moduli (G′ and G″), crosslinker concentration, and cell density. Process descriptors may include nozzle diameter, extrusion pressure, printing speed, temperature, crosslinking mode, light dose, ionic environment, and support-bath conditions.

Dynamic hydrogel behavior requires additional representations. Time-dependent descriptors may include gelation onset time, gelation half-time, gelation rate, plateau modulus, post-shear recovery at defined time points, recovery half-time, hysteresis-loop area, swelling ratio over time, degradation rate, degradation half-life, and self-healing recovery rate. Stimulus-response descriptors may include stimulus type, stimulus threshold, response amplitude, response time, reversibility, fatigue across repeated cycles, temperature, pH, light dose, ionic concentration, enzyme concentration, and state-transition boundaries. These properties can be encoded as summary metrics, multiple time-point vectors, full time-series curves, parameters fitted from kinetic models, or physics-informed features. Model outputs may include rheological properties, gelation behavior, printability, mechanical stability, and biological outcomes such as cell viability and maturation. Recent studies show that machine learning can predict printability, rheological behavior in hybrid hydrogels, extrusion-relevant bioink properties, and as-extruded cell viability, indicating that both physicochemical and biological endpoints are becoming tractable targets (Chen et al., 2023; Deng et al., 2025; Sarah et al., 2025). However, direct AI applications targeting time-dependent behaviors of smart hydrogel bioinks remain scarce.

Together, progress in AI-assisted bioink design will depend not only on algorithms, but also on how hydrogel systems are digitized, standardized, and computationally represented. More interoperable datasets, improved reporting standards, and feature representations that better capture the coupling among formulation, printing, and function will be essential for progress (Wang et al., 2025; Ramesh et al., 2024).

### 3.2 Methodological taxonomy for AI-assisted smart hydrogel bioink development

Several AI methodologies are relevant to smart hydrogel bioink development, but they should not be treated as interchangeable modules (Ramesh et al., 2024; Wang et al., 2025). Supervised learning predicts predefined outcomes from labeled examples, such as viscosity, yield stress, storage or loss modulus, strand width, printability class, pore fidelity, positional error, or cell viability (Chen et al., 2023; Oh et al., 2023; Zhang C. et al., 2024). It is therefore a prediction strategy, not an optimization or control strategy. Bayesian optimization is an objective-directed search strategy that uses a surrogate model and an acquisition function to propose formulations or printing conditions expected to improve a predefined objective, such as a printability score or multi-objective utility (Xu et al., 2024). Because candidate conditions are typically evaluated across successive experimental runs, it is generally a between-run optimization approach rather than a form of real-time closed-loop control. Its primary function is objective-directed experimental search, in which exploitation of promising conditions is balanced with exploration of uncertain regions. Active learning selects new samples that are expected to provide high information gain, reduce model uncertainty, or improve coverage of a sparse design space (Lookman et al., 2019). Its primary function is information-oriented sample selection rather than immediate objective maximization.

Feedback control is defined here more narrowly than iterative optimization. It requires real-time sensing, automated analysis or state estimation, automated decision-making, online adjustment of printing parameters or tool paths, and repeated in-process feedback within the same fabrication process. By contrast, post-print evaluation followed by next-round parameter revision is better described as feedback-informed optimization or between-run iterative learning. AI agents are workflow coordination tools that may support literature retrieval, experimental planning, tool selection, model execution, data interpretation, and result summarization. They are not physical controllers and do not automatically constitute closed-loop bioprinting. Table 2 summarizes these methodological boundaries, required inputs, outputs, validation standards, evidence status, and key limitations.

**TABLE 2:** Methodological taxonomy of AI-assisted approaches for smart hydrogel bioink development and 3D bioprinting.

AI methodology	Primary objective	Required data or typical inputs	Typical outputs	Validation standard	Evidence status	Key limitations
Supervised learning	Predict predefined outcomes from labeled data	Formulation descriptors, rheology, printing settings, images, and biological labels	Viscosity, yield stress, storage/loss modulus, strand width, printability class, pore fidelity, positional error, or cell viability	Grouped splits and external testing matched to the intended generalization level; regression, classification, or segmentation metrics as appropriate	Directly demonstrated for several bioink and bioprinting prediction tasks, including smart hydrogel-relevant endpoints	Requires representative labels; can leak correlated samples; does not optimize or control by itself
Bayesian optimization (BO)	Search for formulations or printing conditions that improve a predefined objective	Initial experimental data, surrogate model, acquisition function, objective function, and feasible parameter bounds	Next candidate formulation, parameter set, or process window	Prospective experimental confirmation of proposed candidates and comparison with random, grid, or expert-guided search	Demonstrated in broader materials and bioprinting contexts; smart hydrogel bioink-specific evidence is emerging	Usually between-run optimization; objective choice can be narrow; does not equal real-time feedback control
Active learning (AL)	Select the most informative new samples to improve model coverage or reduce uncertainty	Unlabeled candidate pool, current model, uncertainty or diversity criterion, and experimental budget.	Priority list of experiments for model improvement or domain coverage	Improved calibration, reduced uncertainty, and maintained performance on held-out groups after added experiments	Useful methodological strategy; direct smart hydrogel bioink applications remain limited	May not select the best-performing candidate; depends on uncertainty quality and feasible experimental design
Transfer learning	Improve data efficiency and portability across related domains	Pretrained model or representation plus target data from a new batch, printer, formulation family, laboratory, or imaging domain	Adapted model, transferred descriptors, or domain-adapted predictions	Target-domain validation, leave-one-domain-out testing, and checks for negative transfer	Adjacent hydrogel/materials examples are increasing; smart bioink-specific validation is still limited	Domain shift can invalidate transferred features; crystal or molecular models may not transfer directly to hydrated polymer networks
Computer vision (CV)	Convert images or videos into quantitative process and construct measurements	Microscopy, camera, video, layer images, segmentation masks, annotations, or tool-path records	Defect labels, strand width, pore geometry, layer registration, cell distribution, construct morphology, or monitoring signals	Dice coefficient, intersection over union (IoU), pixel-level precision/recall, field- or construct-level grouped validation	Well supported in broader bioprinting and increasingly relevant to smart hydrogel bioinks	Image bias, annotation variability, imaging modality shifts, and laboratory-specific settings limit portability
Feedback control	Adjust printing parameters or tool paths online during the same fabrication process	Real-time sensing, state estimation, decision logic, actuator access, and repeated in-process measurements	Online pressure, speed, path, temperature, light, or extrusion adjustments	Demonstrated in-process correction, stability under perturbation, and comparison with open-loop printing	Mostly broader extrusion-bioprinting/manufacturing evidence; direct smart hydrogel bioink evidence remains limited	Requires reliable sensing, low-latency decisions, sterile-compatible integration, and validated safety constraints
AI agents	Coordinate software tools and experimental workflow steps	Literature, protocols, datasets, model outputs, tool APIs, user goals, and experimental constraints	Plans, retrieved evidence, executable model steps, summaries, or suggested experiments	Human-audited accuracy, traceability, tool-use reliability, and validation of downstream experimental decisions	Adjacent-field inspiration from drug discovery, materials science, and robotic chemistry; direct smart bioink evidence is limited	Not physical controllers; cannot replace sensing or automation; autonomous bioink laboratories remain prospective

Abbreviations: AI, artificial intelligence; BO, Bayesian optimization; AL, active learning; CV, computer vision; IoU, intersection over union.

Transfer learning aims to improve data efficiency and portability by adapting representations or model parameters across batches, printers, material systems, laboratories, or related domains, but it does not indicate a higher level of automation and may fail under domain shift or negative transfer (Bracco et al., 2025). Computer vision should be treated as an image-derived quantification method that converts printing or construct images into measurable structural, cellular, and process descriptors (Strauß et al., 2021; Zanderigo et al., 2025).

The evidence supporting these methods also differs. Direct smart hydrogel bioink evidence is strongest for supervised prediction, printability assessment, offline optimization, and image-based analysis (Chen et al., 2023; Xu et al., 2024; Zanderigo et al., 2025). Evidence for feedback control is stronger in broader extrusion-bioprinting or manufacturing contexts than in smart hydrogel bioink-specific systems (Kelly et al., 2025; Bu et al., 2026). Foundation models, AI agents, robotic chemistry, self-driving laboratories, and autonomous workflows provide useful methodological inspiration from adjacent fields, but they remain prospective concepts for smart hydrogel bioink discovery unless they are validated in hydrogel-specific formulation, printing, culture, and functional testing workflows (Moro et al., 2025; Song et al., 2025; Liu et al., 2026). This taxonomy is intended to link each method to a concrete experimental question. Supervised models ask whether a measured endpoint can be predicted for a candidate formulation or process setting. Bayesian optimization asks which candidate should be tested next to improve an objective. Active learning asks which experiment would most improve the model. Transfer learning asks whether knowledge from one material, printer, laboratory, or imaging domain remains valid in another. Computer vision asks how printed structures and cells can be quantified from images. Feedback control asks whether measurements can drive online correction during fabrication. AI agents ask how computational tasks can be coordinated, audited, and documented. Treating these questions separately helps prevent prediction accuracy from being mistaken for optimization, monitoring from being mistaken for control, or software coordination from being mistaken for physical autonomy.

### 3.3 AI-assisted prediction of bioink properties, formulation-printability relationships, and biological performance

AI can predict formulation-dependent properties before extensive experimental validation, estimating whether a candidate formulation shows suitable rheological behavior, favorable printability potential, and acceptable biological performance. Existing or relatively direct applications include prediction of viscosity, shear stress, printability classification, printing resolution, positional error, and as-extruded cell viability (Chen et al., 2023; Zhang C. et al., 2024; Oh et al., 2025). These tasks are valuable because they link formulation descriptors and process variables to measurable outcomes that can be collected in current bioprinting workflows. Recent studies have shown that machine learning can predict viscosity and shear-stress behavior of hyaluronic acid methacrylate (HAMA)/GelMA hybrid hydrogels from formulation variables, and related approaches have been applied to alginate/gelatin/nanocellulose bioinks across shear-rate conditions (Sarah et al., 2025; Deng et al., 2025).

More prospective targets include gelation time, gelation trajectory, post-shear recovery, self-healing efficiency, swelling trajectory, degradation trajectory, stimulus-response threshold, stimulus-induced state transition, reversibility, and response fatigue. These targets are particularly important for smart hydrogel bioinks, but direct AI applications targeting time-dependent behaviors remain scarce. Machine learning can also predict biological performance, because a formulation with promising rheological properties may still be biologically suboptimal. Current evidence includes models for as-extruded cell viability and early-stage material predictors of neural bioink performance, but many studies remain limited to narrow material systems (Zhang C. et al., 2024; da Silva et al., 2026). Prediction tasks therefore remain context-dependent, and model transferability across bioinks, support baths, printers, and biological systems is limited. The evidence is strongest for specific prediction tasks in broader bioink or bioprinting contexts, while many smart-bioink-specific dynamic properties should be framed as emerging or prospective targets rather than mature applications.

### 3.4 Foundation models for material property representation and transfer learning in bioink contexts

The recent emergence of foundation models in chemistry and materials science introduces a new paradigm for representation learning that may offer methodological inspiration for bioink research, although direct application to hydrated polymeric systems remains limited. Many crystal-domain foundation models are trained on periodic structures, relatively well-defined atomic environments, crystalline inorganic solids, and static or near-equilibrium structural representations. Graph neural network models such as M3GNet, MEGNet, and CHGNet have been pre-trained on large crystal structure databases, learning interatomic potential functions and property descriptors that transfer well to crystalline inorganic solids but may not directly capture the physics of hydrated polymer networks (Ko et al., 2025; Yuan et al., 2026).

Hydrogel bioinks differ substantially from these source domains. They are amorphous, highly hydrated polymer networks governed by solvent-mediated interactions, polymer-chain conformation, entanglement, reversible and dynamic crosslinking, swelling, degradation, processing and shear history, additives, embedded cells, bioactive components, cell-mediated remodeling, mesoscale network structure, and time-dependent properties. Crystal-structure representations may therefore fail to encode key determinants of bioink behavior, creating substantial domain shift and possible negative transfer. Polymer-specific or hydrogel-specific pretraining may be more appropriate than direct use of crystal models, but this remains to be demonstrated.

**FIGURE 3:**
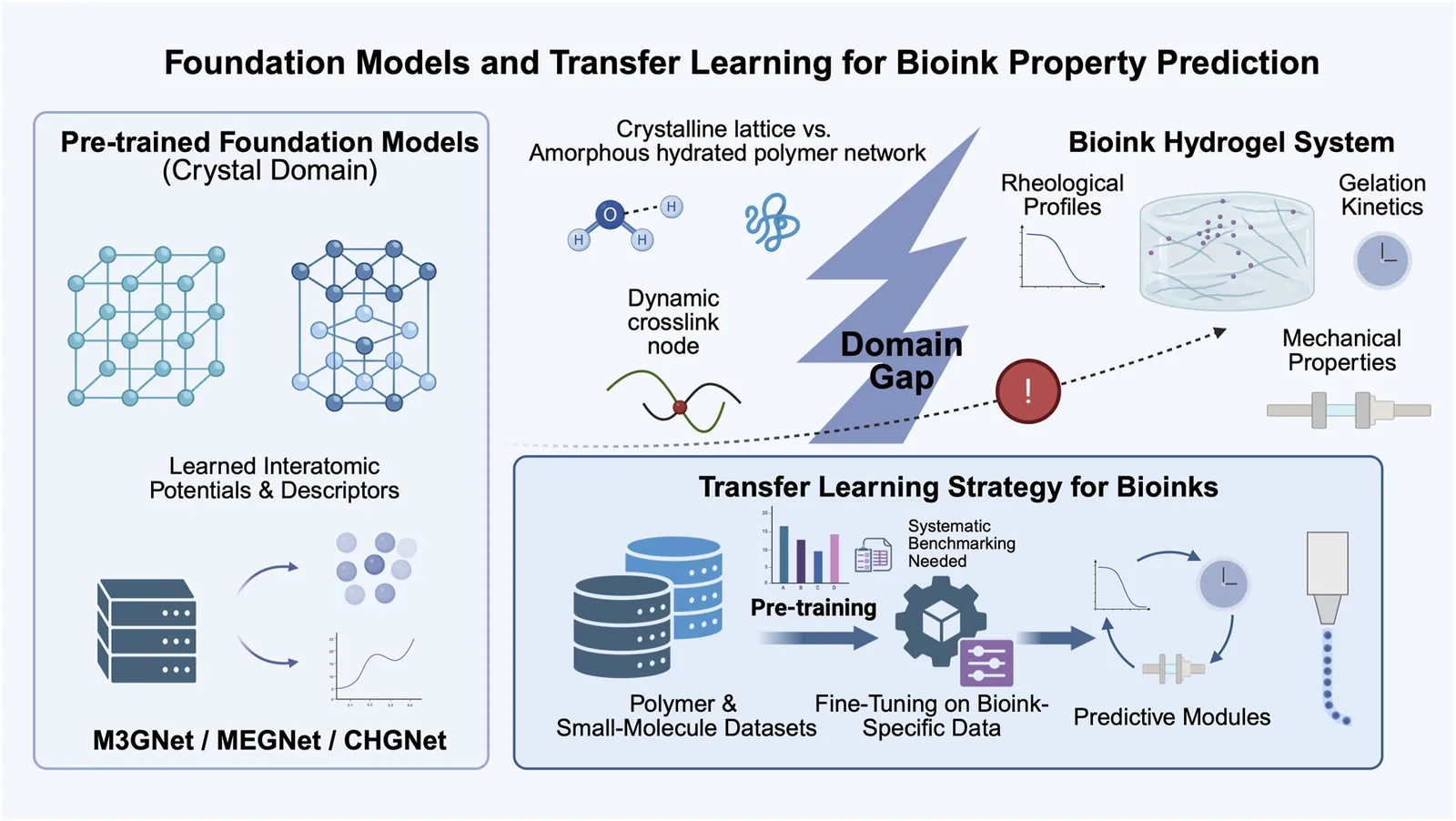
Foundation models and transfer learning for bioink property prediction. General-purpose models from molecular, materials, imaging, or biological domains may provide useful representations, but direct transfer to hydrated polymer networks requires hydrogel-specific benchmarking. Domain shift, negative transfer, limited bioink datasets, and endpoint mismatch should be evaluated before these models are used for formulation or biological predictions. Created in BioRender. Luo, Y. (2026) https://BioRender.com/73behfm.

Transfer learning can involve transfer of feature representations, transfer of model parameters, adaptation across batches, printers, material systems, laboratories, or adaptation from polymer datasets to bioink-specific datasets. A recent bioprinting study suggests that transfer learning can reduce retraining demands across experimental settings (Bracco et al., 2025), but transfer learning should not be treated as a separate automation level. It is a strategy for improving data efficiency and portability. Future transfer-learning models should be compared with simple baselines, including linear models, random forests, gradient boosting, and physically informed handcrafted descriptors. This review does not perform new benchmark experiments; rather, systematic benchmarking is identified as an important future need. Figure 3 illustrates the domain gap and summarizes a cautious route from polymer or small-molecule datasets to bioink-specific property prediction.

For smart hydrogel bioinks, the key question is therefore not whether a large model or transferred representation is technically available, but whether its learned features correspond to the physicochemical and biological variables that control bioink behavior (Bracco et al., 2025; Moro et al., 2025; Yuan et al., 2026). A crystal-domain model may encode local atomic environments well while missing polymer hydration, network heterogeneity, ionic interactions, dynamic crosslinking, shear recovery, degradation, or cell-mediated remodeling (Ko et al., 2025; Hölzl et al., 2016; Theus et al., 2020; Neumann et al., 2023). Similarly, an image model trained on one microscope, annotation protocol, or construct geometry may not transfer to a different laboratory without calibration (Strauß et al., 2023; Grijalva Garces et al., 2023; Bradshaw et al., 2023). Hydrogel- or polymer-specific pretraining, explicit domain-adaptation tests, negative-transfer checks, out-of-distribution assessment, and grouped external validation are therefore needed before foundation models or transfer learning can be used as evidence for bioink design decisions.

## 4 AI for printability prediction and process optimization

### 4.1 Defining and quantifying printability

Printability prediction is central to AI-assisted hydrogel bioprinting, but printability should be treated as a layered outcome rather than a single universal label (Gillispie et al., 2020; Schwab et al., 2020). Extrusion-level printability concerns flow initiation, pressure stability, shear exposure, and nozzle compatibility (Paxton et al., 2017; Fu et al., 2021). Filament-level printability concerns strand width, continuity, spreading, fusion, and collapse, whereas construct-level printability concerns pore fidelity, layer registration, shape retention, positional error, and structural stability (Gillispie et al., 2020; Schwab et al., 2020). Biological compatibility and function-related outcomes include immediate viability, proliferation, phenotype maintenance, differentiation, extracellular matrix deposition, maturation, and tissue-specific function (Dubbin et al., 2017; Theus et al., 2020). A formulation may satisfy one layer while failing another, so AI models should report the layer being predicted and the measurement protocol used.

This layered definition also affects how data should be curated and benchmarked. A model trained on extrusion pressure or filament width cannot be assumed to predict construct stability or biological compatibility. Likewise, an image-based pore-fidelity model does not establish that encapsulated cells maintain phenotype or mature into a functional tissue. For this reason, printability datasets should report formulation composition, polymer concentration and molecular weight, degree of functionalization where relevant, crosslinking conditions, nozzle geometry, pressure or speed, environmental conditions, imaging protocol, and endpoint definitions (Strauß et al., 2023; Grijalva Garces et al., 2023). Without this metadata, apparent model performance may reflect laboratory-specific conventions rather than transferable printability principles. Recent work suggests a shift toward more objective and data-rich frameworks. Standardization efforts have emphasized controlling extrusion rate and accounting for bioink-specific flow behavior to improve inter-study comparability. At the same time, image-based analytics and deep learning-assisted similarity analysis are beginning to provide more automated ways to assess how closely printed structures match expected geometries. These developments are especially important for AI applications, which require measurable, reproducible, and machine-readable outputs rather than subjective descriptions alone (Strauß et al., 2021; Strauß et al., 2023; Balters and Reichl, 2025). Despite this progress, no single metric can fully capture printability across different bioinks, printers, and application settings (Gillispie et al., 2020; Schwab et al., 2020; Fu et al., 2021). A formulation optimized for smooth extrusion may still show poor pore fidelity, whereas conditions that improve structural fidelity may also affect cell viability (Schwab et al., 2020; Fu et al., 2021). Printability is therefore best treated as a composite performance domain rather than a universal scalar variable. Clearer quantitative descriptors and more standardized evaluation workflows remain essential for AI-assisted modeling of printability outcomes.

### 4.2 Printability prediction, computer vision, and monitoring

Once printability is defined by measurable descriptors, AI can model it as a material-process-outcome mapping problem. Supervised prediction uses formulation features such as composition and rheology, together with process variables including nozzle diameter, extrusion pressure, printing speed, and temperature, to predict filament width, strand continuity, pore fidelity, layer stacking quality, printing resolution, positional error, or printability class (Oh et al., 2023; Oh et al., 2025). In this context, computer vision has a distinct role because it converts images or videos generated during or after printing into quantitative descriptors of print quality. These descriptors may include filament segmentation, strand-width measurement, pore analysis, defect detection, layer registration, tool-path deviation, cell-distribution analysis, construct characterization, and process-monitoring readouts. These image-derived measurements can serve as labels for supervised prediction models, input features for quality assessment, or signals for monitoring systems.

Image-based and sensing-enhanced modeling is valuable because rheology alone often cannot fully capture printing behavior. Optical, thermal, and geometry-based assessment can provide richer descriptions of strand morphology and print quality (Strauß et al., 2021; Strauß et al., 2023; Gugliandolo et al., 2024; Balters and Reichl, 2025). Real-time monitoring refers to acquiring and analyzing images, pressure, temperature, geometric, or other process data during printing. It may detect deviations or defects during fabrication, but it does not necessarily change the process. Computer vision becomes part of feedback control only when its outputs are automatically used to modify printing parameters, tool paths, or other process variables during fabrication. Camera imaging, defect classification, or real-time display should therefore be described as monitoring or decision support unless automated in-process correction is present.

Overall, AI-assisted printability modeling is shifting assessment from retrospective evaluation toward prospective prediction and quantitative monitoring. By estimating likely outcomes before or alongside experimentation, AI can narrow the search space, reduce empirical trial-and-error, and make printability assessment more reproducible. However, evidence should be interpreted according to the level of automation demonstrated: supervised prediction, image-based quantification, real-time monitoring, and feedback control are related but distinct.

### 4.3 Offline parameter optimization and process-window identification

Once printability outcomes can be modeled quantitatively, AI can be used to identify favorable printing conditions before printing begins. In extrusion-based bioprinting, key variables include extrusion pressure, printing speed, nozzle diameter, temperature, deposition spacing, and crosslinking-related settings. Offline parameter optimization selects these settings before fabrication and remains an open-loop process because the system does not automatically change parameters in response to deviations during printing. Bayesian optimization can be useful for formulation optimization, parameter selection, and process-window identification, especially when each printing trial requires substantial time, materials, and biological resources. Ruberu et al. combined a quantitative printability score with Bayesian optimization to identify favorable extrusion-printing conditions for GelMA- and HAMA-based bioinks while reducing experimental burden (Ruberu et al., 2021). This is feedback-informed iterative optimization across trials, not necessarily real-time adaptive control.

Optimization does not always aim to find a single best parameter point. In practical printing settings, robustness can be as important as peak performance. AI can help identify process windows, namely parameter regions where acceptable printability, geometric fidelity, and biological outcomes can be achieved reproducibly. Multi-objective optimization is also important because conditions that improve strand fidelity or structural precision may increase shear stress and reduce cell survival, while conditions that support cell viability may lower resolution or weaken shape retention. Recent work suggests that machine learning-based frameworks can predict and optimize biologically relevant outcomes such as as-extruded cell viability, supporting the view that process optimization should consider both manufacturability and biological function (Zhang C. et al., 2024). Several challenges still need to be addressed. Many optimization studies are based on limited material systems or restricted printing settings, which restricts transferability across different bioinks, printers, and application contexts (Yu et al., 2025). The results also depend strongly on how the target function is defined. A model optimized only for filament width or shape fidelity may not perform well when biological criteria, such as cell viability or long-term function, are added. Further progress will require stronger benchmark datasets, clearer optimization objectives, and broader validation across materials, printers, and cell systems.

### 4.4 Levels of automation in AI-assisted bioprinting

AI-assisted bioprinting should be described using explicit levels of automation. Level 1 is offline parameter optimization, in which parameters are selected before printing begins and do not change automatically during fabrication. This is an open-loop process. Level 2 is real-time monitoring, in which images, pressure, temperature, geometry, or other process data are acquired and analyzed during printing. Monitoring can report deviations or defects, but automated correction is not required. Level 3 is feedback control, which must include real-time sensing, automated analysis or state estimation, automated decision-making, parameter or tool-path adjustment, and repeated feedback during the same printing process. Level 4 is a fully autonomous laboratory, which would also integrate formulation preparation, experiment planning, sample selection, model updating, printing, construct culture or maturation, post-print evaluation, and next-round decision-making with limited human intervention.

These levels are not equivalent and are not equally mature. Thermal imaging strategies and modular *in situ* monitoring platforms show that print quality can be quantified during fabrication rather than only after printing is complete (Gugliandolo et al., 2024; Zanderigo et al., 2025). These studies support real-time monitoring and decision support. More direct evidence for feedback correction includes camera-based monitoring with neural networks for extrusion quality classification and automated error correction, vision-based tool-path compensation, and uncertainty-aware real-time quality correction (Kelly et al., 2025; Barjuei et al., 2024; Bu et al., 2026). These emerging strategies have also been summarized in recent reviews of AI-driven closed-loop bioprinting (Ke, 2026). Such examples indicate movement from monitoring toward in-process regulation, but they should not be generalized to all AI-assisted bioprinting studies.

Closed-loop bioprinting should therefore be reserved for systems that repeatedly use real-time process information to update printing actions during fabrication. Printing results used after a run to guide the next experiment are better described as between-run iterative learning, feedback-informed optimization, or iterative model updating. For smart hydrogel bioinks, real-time feedback control is attractive because material behavior can shift with temperature, shear history, crosslinking, and time. Nevertheless, fully integrated autonomous bioink laboratories remain prospective, because they would require reliable sensing, sterile-compatible automation, model updating, construct maturation, functional testing, and next-round experimental planning. To clarify these distinctions, Table 3 summarizes representative AI tasks across the smart hydrogel bioprinting workflow.

**TABLE 3:** Representative AI tasks across the smart hydrogel bioprinting workflow.

Workflow task or stage	Typical methods	Typical outputs	Practical value	Representative references
Data representation	Feature engineering and multimodal integration	Structured material-process-function datasets	Builds the basis for predictive modeling	Wang et al. (2025), Ramesh et al. (2024)
Formulation screening	Supervised learning	Printable/non-printable classification and candidate ranking	Reduces empirical trial and error	Chen et al. (2023), Zhou et al. (2025)
Multi-objective formulation design	Surrogate modeling and Pareto-oriented optimization	Trade-off regions and preferred candidate formulations	Balances printability, viability, and mechanics	Liu et al. (2025), Jiang et al. (2025)
Sequential experimental optimization	Bayesian optimization and surrogate-assisted search	Suggested next experiments or process windows	Improves screening efficiency before printing or between printing runs	Ruberu et al. (2021), Xu et al. (2024)
Active learning-based sample selection	Model-in-the-loop sample selection	Informative candidates and updated models	Reduces experimental burden under limited throughput	Wang et al. (2025), Zhou et al. (2025)
Inverse or generative design	Inverse design, generative modeling, or recommendation-oriented AI	Candidate formulations matching target profiles	Shifts from forward prediction to target-oriented design	Zhou et al. (2025), Jin et al. (2026)
Rheology prediction	Regression-based ML models	Viscosity, shear-thinning behavior, and recovery behavior	Predicts extrusion-relevant behavior early	Sarah et al. (2025), Deng et al. (2025)
Material-property-to-printability mapping	Hierarchical ML and property prediction	Printability-related potential and formulation stability	Connects intrinsic material features with likely printability	Geevarghese et al. (2025), Oh et al. (2025)
Biological outcome prediction	Integrated ML frameworks	As-extruded cell viability and early biological response	Introduces biological relevance into screening	Zhang C. et al. (2024), da Silva et al. (2026)
Printability and shape-fidelity modeling	Rheology-informed and image-based ML	Resolution, pore fidelity, strand width, and positional error	Makes printability measurable and predictable	Oh et al. (2023), Balters and Reichl (2025)
Process parameter optimization	Bayesian optimization and data-driven search	Optimal settings or robust process windows	Replaces manual one-factor-at-a-time tuning	Ruberu et al. (2021), Zhang Q. et al. (2025), Yu et al. (2025)
Real-time process monitoring	Image segmentation, CNNs, and vision analytics	Defect detection and quality classification	Supports process-aware monitoring but does not automatically correct errors by itself	Gugliandolo et al. (2024), Zanderigo et al. (2025)
Feedback control	Vision-guided compensation and uncertainty-aware correction	Online adjustment of pressure, speed, temperature, or tool path	Supports in-process correction when automated feedback is present	Barjuei et al. (2024), Bu et al. (2026)
Post-print characterization and quality assessment	Computer vision, segmentation, classification, and multimodal analysis	Construct-level quality metrics and post-print structural assessment	Extends AI analysis from printing conditions to final construct quality	Spiller and Duarte Campos (2025), Silva Robazzi et al. (2025), Perin et al. (2026)
Early biological outcome prediction	Supervised learning and integrated prediction models	Early cell viability and selected biological-response estimates	Extends screening beyond purely rheological and geometric endpoints	Zhang C. et al. (2024), da Silva et al. (2026)

Abbreviations: AI, artificial intelligence; ML, machine learning; CNN, convolutional neural network.

This distinction is especially important when interpreting studies that combine monitoring, prediction, and iterative optimization. Filament-defect detection during printing mainly provides monitoring evidence. Recommendations of pressure or speed for the next run represent feedback-informed refinement. By contrast, automatic adjustment of pressure, speed, temperature, light exposure, or tool path during the same run provides stronger evidence for feedback control. These categories differ in hardware requirements, latency constraints, safety risks, and validation standards. For smart hydrogel bioinks, the distinction is amplified by time-dependent gelation, stimuli responsiveness, cell sensitivity, and batch-to-batch variability. Claims of closed-loop or autonomous performance should therefore specify the sensing modality, decision rule, actuator, update frequency, process variable adjusted, and whether correction occurred during fabrication or between experiments.

### 4.5 AI agents and workflow coordination

**FIGURE 4:**
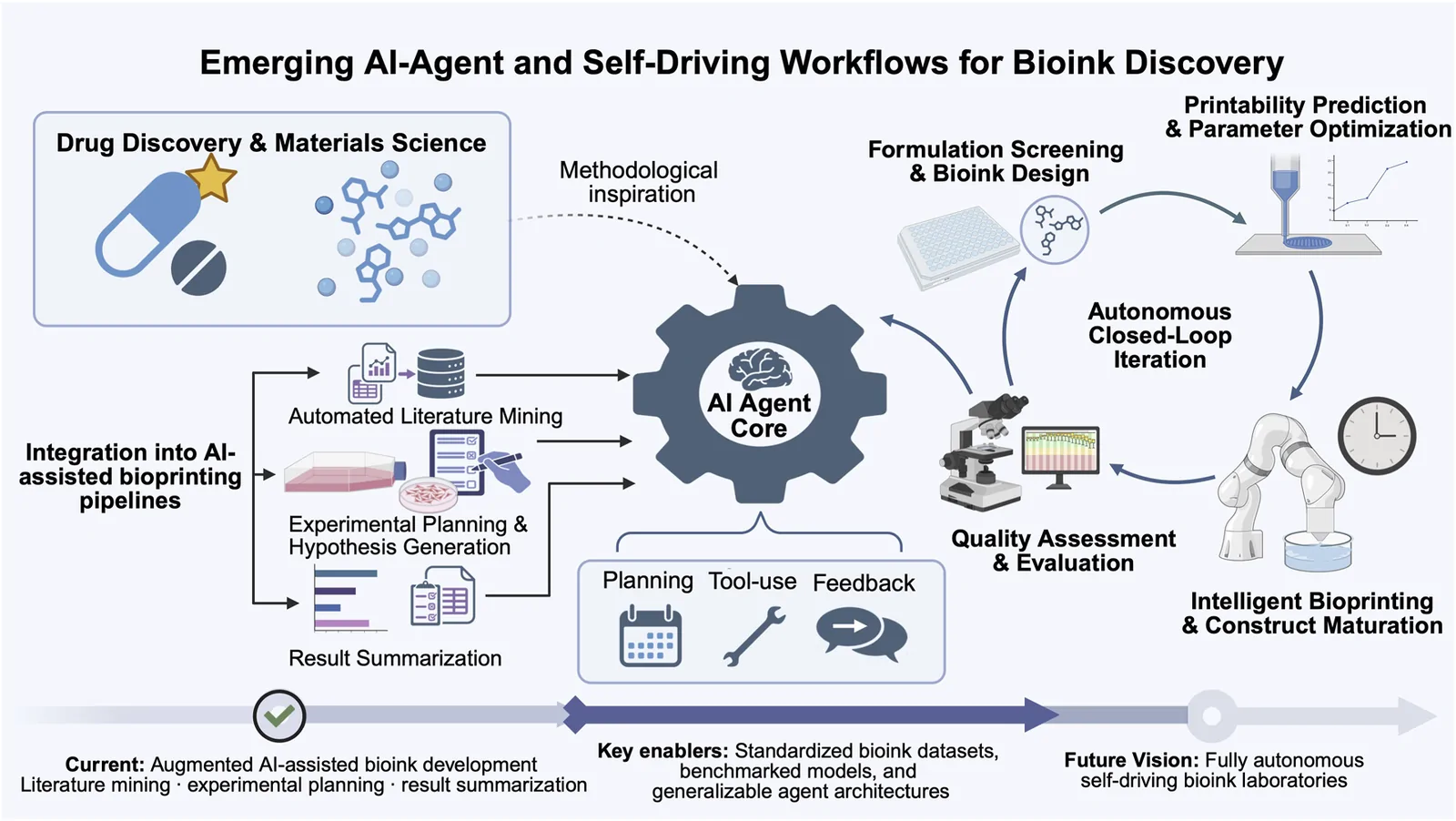
Emerging AI-Agent and Self-Driving Workflows for Bioink Discovery. Current agent use should be interpreted as assistance for planning, literature retrieval, tool selection, model execution, and result summarization. Examples from drug discovery, materials science, and robotic chemistry provide methodological inspiration rather than direct evidence in smart hydrogel bioink discovery. Fully autonomous self-driving bioink laboratories remain a future vision requiring validated endpoints, integrated automation, robust sensing, external validation, and human oversight. Created in BioRender. Luo, Y. (2026) https://BioRender.com/6wv61y2.

AI agents coordinate information and software tasks across the experimental workflow rather than regulating the physical printing process. Figure 4 summarizes the potential roles of AI agents in workflow coordination and distinguishes current assisted functions from prospective autonomous bioink discovery. They may assist literature retrieval, protocol drafting, experimental planning, model execution, tool selection, data interpretation, result summarization, and workflow documentation. However, agent-assisted workflow coordination is not equivalent to feedback control: agents are not physical controllers, cannot replace real-time sensing or actuator-level control, and do not by themselves constitute closed-loop bioprinting systems.

Direct evidence for AI agents in smart hydrogel bioink discovery remains limited. Multi-agent systems, robotic AI chemists, and self-driving bioprinting laboratories provide adjacent-field methodological inspiration from drug discovery, materials science, chemistry, and biofabrication, but they do not yet validate autonomous bioink preparation, printing, maturation, and functional testing (Song et al., 2025; Liu et al., 2026). A self-driving bioink laboratory therefore remains a future vision that would require standardized datasets, validated biological and functional endpoints, sterile-compatible automation, reliable formulation preparation, integrated printing and culture systems, robust sensing, model calibration, external validation, human oversight, and regulatory traceability.

Near-term uses are more appropriately framed as assisted coordination and documentation (Salas et al., 2026; Liu et al., 2026). An agent may retrieve hydrogel formulations, summarize rheological ranges, run a trained prediction model, compare candidate process windows, document provenance, or prepare a structured plan for human review. These functions may reduce cognitive burden and improve traceability, but experimental validation remains decisive before any suggested formulation is fabricated or biologically tested.

## 5 AI for construct characterization, validation, and quality assurance

**FIGURE 5:**
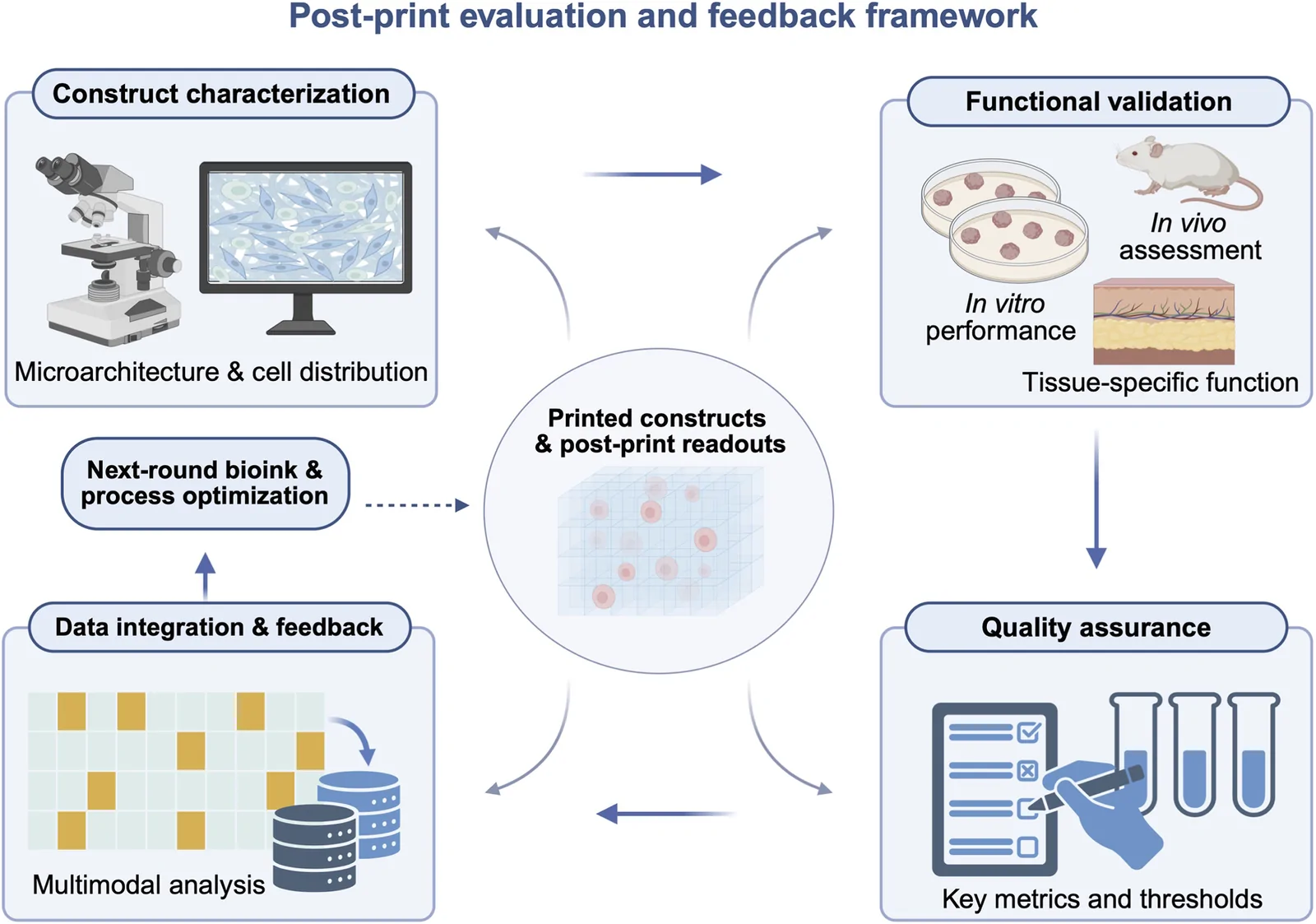
Post-print evaluation and feedback framework. Structural, cellular, and functional readouts generated after printing can support next-round bioink and process optimization, model updating, quality assurance, and reproducibility assessment. This between-run feedback should not be equated with real-time closed-loop control unless automated in-process measurements directly drive online parameter adjustment. Created in BioRender. Luo, Y. (2026) https://BioRender.com/cpxq0sz.

At this stage, the focus shifts from fabrication accuracy to the reproducible characterization, functional validation, and quality assessment of printed constructs. Recent reviews have identified post-printing assessment, construct maturation, and quality assurance as essential steps for advancing AI-assisted bioprinting toward reliable tissue engineering applications (Spiller and Duarte Campos, 2025; Perin et al., 2026). Figure 5 summarizes the post-printing evaluation and feedback framework discussed in this section. It shows how construct characterization, functional validation, quality assurance, and data feedback can support iterative refinement of both bioink design and printing parameters. Such feedback is best described as between-run learning unless it automatically changes printing actions during fabrication.

### 5.1 AI-assisted post-printing construct characterization

Post-printing construct characterization is essential because successful fabrication cannot be assessed solely by extrusion behavior or shape fidelity during printing. Recent analyses of 3D-bioprinted tissues have highlighted the lack of standardized success criteria for finished constructs and the need for more advanced imaging and analysis methods to ensure reproducible characterization (Spiller and Duarte Campos, 2025). Therefore, printed constructs should be evaluated at multiple levels, including structural integrity, geometric accuracy, pore architecture, cell distribution, cell viability, matrix formation, and tissue maturation.

AI is increasingly valuable at this stage because post-printing characterization often generates large and complex datasets that are difficult to analyze manually. Data from bright-field microscopy, fluorescence imaging, confocal microscopy, optical coherence tomography, micro-CT, and other multimodal workflows can be analyzed using AI-assisted segmentation, feature extraction, defect recognition, spatial quantification, and pattern classification. Post-printing assessment is shifting from qualitative inspection toward more automated and quantitative characterization (Silva Robazzi et al., 2025; Spiller and Duarte Campos, 2025). From a structural perspective, AI can help quantify whether printed constructs retain their intended geometry after crosslinking and culture, using descriptors such as filament diameter, pore size, connectivity, layer registration, volumetric shrinkage or swelling, and spatial deviation from the design model. Biological characterization can further extend beyond live/dead assays to include cell distribution, density heterogeneity, spheroid morphology, tissue-specific marker expression, extracellular matrix deposition, and signs of maturation over time. AI is therefore useful for linking structural and biological readouts in ways that are difficult to achieve through manual scoring alone (Lee et al., 2024; Liu et al., 2026). Another advantage of AI-assisted characterization is its compatibility with longitudinal analysis. Printed constructs continue to change after fabrication through cell proliferation, matrix remodeling, contraction, degradation, and maturation. Static endpoint assessment may therefore miss important temporal changes. AI-based pipelines can support repeated and time-resolved analysis of construct development (Filippi et al., 2025; Liu et al., 2026). In this way, post-printing observations can become part of an iterative learning cycle rather than passive end-stage measurements.

Despite its promise, AI-assisted post-printing characterization still faces several challenges. Imaging quality, annotation standards, segmentation accuracy, and biological endpoint definitions vary widely across platforms and tissue models, which can limit reproducible analysis (Spiller and Duarte Campos, 2025). A construct that appears geometrically accurate may still fail biologically. Conversely, a biologically active construct may deviate from its original design because of remodeling or maturation. These issues indicate that characterization should not rely on a single metric. Instead, structural, cellular, and functional information should be integrated within a more standardized analytical and quality-oriented framework (Perin et al., 2026).

### 5.2 AI-assisted functional assessment and validation of printed constructs

Functional validation is a necessary step after post-printing characterization because a construct that appears geometrically accurate or structurally stable is not necessarily biologically meaningful (Murphy and Atala, 2014; Matai et al., 2020). Immediate post-print viability is necessary but insufficient. Evaluation should be time-resolved, spanning the immediate post-print phase, early culture phase, intermediate remodeling phase, and late maturation phase (Derakhshanfar et al., 2018; Mandrycky et al., 2016). Relevant endpoints may include proliferation, apoptosis, phenotype maintenance, migration, differentiation, extracellular matrix deposition, matrix remodeling, tissue maturation, tissue-specific function, and response to perturbation or treatment. AI-assisted analysis can help extract quantitative and biologically meaningful information from multidimensional imaging, phenotype, and functional datasets, but the endpoints must be aligned with the intended use of the construct rather than reduced to a universal viability score.

This broader view is particularly important because geometric fidelity and immediate post-print viability are necessary but insufficient indicators of tissue engineering success (Dubbin et al., 2017; Schwab et al., 2020; Hull et al., 2022). AI-assisted bioink design should progressively connect early material and process descriptors to later structure-function relationships, including proliferation, phenotype maintenance, differentiation, extracellular matrix (ECM) deposition, matrix remodeling, tissue maturation, tissue-specific function, long-term stability, drug response, and inflammatory or immune-relevant responses where applicable (Theus et al., 2020; Kim, 2023; Jiang et al., 2025). The relevant endpoints depend on the intended application. Vascular constructs require lumen formation, perfusability, barrier function, endothelial alignment, vessel stability, and response to flow (Kim and Cho, 2024; Konwarh, 2025; Jäger et al., 2026). Neural constructs require neurite extension, neural lineage marker expression, synaptic marker expression, network activity, and electrophysiological maturation (Li S. et al., 2025; da Silva et al., 2026). Cartilage, bone, and musculoskeletal constructs require matrix deposition, collagen or glycosaminoglycan production, mineralization when relevant, mechanical maturation, and load-bearing behavior (Galarraga et al., 2019; Theus et al., 2020; Jaouher et al., 2025). Tumor, disease, organoid, and personalized testing platforms require drug response, invasion, hypoxia, tumor-stromal interaction, immune or vascular microenvironmental phenotypes, reproducibility, long-term maintenance, batch-to-batch consistency, and preservation of patient-specific phenotypes. Early descriptors such as hydrogel chemistry, polymer concentration, crosslinking density, stiffness, degradation rate, swelling, shear-thinning behavior, recovery kinetics, nozzle size, pressure, and strand geometry should therefore be linked to later functional readouts when sufficient data are available (Hölzl et al., 2016; Paxton et al., 2017; Fu et al., 2021; Schwab et al., 2020; Oh et al., 2023; da Silva et al., 2026). A printability classifier is most useful when it becomes part of a broader evidence chain from formulation to process, architecture, cell response, maturation, and application-specific function.

### 5.3 Quality assurance and reproducibility in bioprinting

Quality assurance and reproducibility have become central concerns in bioprinting because successful fabrication cannot be judged solely by isolated printing outcomes or visually acceptable constructs. A printed tissue model may appear adequate in a single experiment yet still show poor repeatability across batches, operators, printers, or laboratories. This challenge is especially relevant in extrusion-based bioprinting, where variability in bioink preparation, rheological behavior, environmental conditions, and machine settings can strongly influence results. Reproducibility is therefore not a peripheral technical issue, but a core requirement for the scientific reliability and translational credibility of bioprinting workflows (Grijalva Garces et al., 2023; Maddalon et al., 2026). Quality assurance should be understood as a multi-level framework rather than a single endpoint check. Relevant dimensions include consistency of bioink preparation, stability of printing performance, structural fidelity of constructs, preservation of cell viability and spatial organization, and reproducibility of downstream biological readouts. Recent discussions increasingly frame these dimensions within broader systems such as Quality by Design, in which critical material attributes, process parameters, and quality attributes are explicitly linked and evaluated in a structured manner. This perspective shifts quality control from a reactive strategy toward a more systematic and design-aware workflow (Zhang et al., 2025b; Wang et al., 2025).

AI can strengthen quality assurance in several ways. It can standardize evaluation by converting heterogeneous outcomes into more consistent quantitative descriptors, identify hidden sources of variability across formulations, process settings, or imaging datasets, and enable earlier detection of deviations that may later compromise construct quality. Recent reviews suggest that automation, digitalization, and predictive methods can improve the robustness, efficiency, and reproducibility of biofabrication workflows while reducing wasteful empirical iteration (Filippi et al., 2025; Liu et al., 2026). The increasing focus on standardization further supports this direction. Recent standardization efforts in 3D bioprinting have emphasized the need for clearer criteria, test methods, and terminology across different stages of the bioprinting workflow. These efforts aim to improve the objective assessment of quality, reproducibility, and safety in constructs produced with cell-containing bioinks (Maddalon et al., 2026). Such developments are important for AI because predictive models and automated quality systems depend on the definitions, labels, and measurement frameworks used to train and evaluate them.

However, several barriers remain. Many studies still assess quality using locally defined metrics, material-specific protocols, or printer-specific setups, making cross-study comparison difficult (Grijalva Garces et al., 2023; Zhang et al., 2025b). Reproducibility problems may also arise from combined variation in bioink formulation, hardware, software control, environmental conditions, and biological assays. These issues limit both the transferability of AI models and the comparability of published findings. Future progress will therefore require closer integration of standardized data capture, AI-assisted analysis, process-aware validation, and benchmark-oriented reporting practices.

### 5.4 Data feedback for iterative bioink and process refinement

A major promise of AI-assisted bioprinting is that post-printing data need not remain passive end-stage observations. Post-printing characterization, functional validation, and quality assurance can inform iterative refinement of both bioink design and process parameters across experimental rounds. These feedback data may include geometric deviation, filament integrity, pore architecture, cell organization, maturation, reproducibility, defect patterns, batch effects, and failure modes. When consistently digitized, they can improve prediction, guide updated formulation choices, and refine future process settings (Tănase et al., 2025; Zhang et al., 2025b). This should be described as feedback-informed optimization or iterative model updating unless the data automatically modify parameters during the same fabrication process. Meaningful data feedback still depends on comparable endpoints, traceable links between formulation, process, and outcomes, and variables that capture both structural and biological quality.

Overall, data feedback can help AI-assisted bioprinting move from isolated prediction and optimization tasks toward more reproducible and application-aligned workflows. The strongest current evidence supports property prediction, printability prediction, offline optimization, image-based monitoring, defect detection, and isolated feedback correction. More integrated systems that combine monitoring with automated parameter adjustment, uncertainty-aware correction, and iterative updating across experimental rounds are emerging. Fully integrated autonomous workflows that include formulation preparation, experiment planning, model updating, printing, maturation, construct evaluation, and next-round decision-making remain prospective.

## 6 Representative applications of AI-assisted smart hydrogel bioprinting

### 6.1 Regenerative tissue engineering

Regenerative tissue engineering is one of the most clinically relevant application domains for AI-assisted smart hydrogel bioprinting. Its goal is not only to print a stable construct, but also to create a living and biomimetic tissue substitute that can support repair, integration, and functional recovery. Recent reviews increasingly describe AI as an enabling layer across biomaterial design, structural planning, process optimization, and post-fabrication evaluation (Bagherpour et al., 2025; Zhang Z. et al., 2024; Dhiman et al., 2026). This shift may move regenerative bioprinting from empirical fabrication toward more rational and application-oriented strategies. Smart hydrogels are especially relevant because they provide dynamically tunable biochemical and mechanical microenvironments, while AI helps manage formulation–process–structure–function relationships. Rather than optimizing printability alone, AI can support the selection of hydrogel systems and printing conditions better matched to tissue-specific regenerative needs. Recent literature increasingly treats hydrogel choice, printed geometry, and biological maturation as coupled design problems rather than isolated variables (Bagherpour et al., 2025; Sojdeh et al., 2026). Representative application scenarios and validation endpoints are summarized in Table 4.

**TABLE 4:** Representative application scenarios of AI-assisted smart hydrogel bioprinting.

Application scenario	Typical goal	Role of smart hydrogels	Role of AI	Typical validation endpoints	Main translational challenge	Representative references
Regenerative tissue engineering	Biomimetic constructs for repair and functional recovery	Provide tunable biochemical and mechanical microenvironments	Assist material selection, structure planning, and process optimization	Shape fidelity, viability, proliferation, differentiation, matrix deposition, tissue-specific organization and function	Tissue integration and clinical translation	Bagherpour et al. (2025), Dhiman et al. (2026)
Soft tissue/neural/dental/*in situ* regeneration	Tissue-specific regenerative constructs	Support tissue-adaptive bioactivity and responsiveness	Match formulation and process settings to tissue needs	Construct stability, cell survival, migration, phenotype maintenance, regenerative morphology, tissue-specific maturation	Personalization and robustness across tissues	Huang et al. (2025), Liang et al. (2025)
*In vitro* disease models	Reconstruct disease-relevant 3D microenvironments	Recreate ECM context and multicellular organization	Support model optimization and phenotype analysis	Spatial architecture, disease phenotype, perturbation response, reproducibility, similarity to native tissue behavior	Standardization and human relevance	Tao et al. (2025), Hua and Gaharwar (2026)
Tumor models	Reconstruct tumor architecture and microenvironment-dependent behavior	Support stromal and ECM interactions	Help analyze treatment response and phenotype patterns	Tumor heterogeneity, stromal interaction, matrix remodeling, therapy response, reproducible phenotype	Model complexity and reproducibility	Kurzątkowska et al. (2025), Piotrowska et al. (2025)
Drug screening	Human-relevant pharmacological testing	Mimic tissue stiffness, transport, and cell-matrix interactions	Enable multiparametric response analysis	Viability, morphology, sensitivity, functional response, reproducibility, predictive validity	Benchmarking and predictive validity	Budharaju et al. (2025), Kurzątkowska et al. (2025)
Personalized testing	Patient-specific therapy-response prediction	Support patient-derived 3D microenvironment reconstruction	Extract patient-specific response patterns	Drug sensitivity, phenotype retention, patient-specific response patterns, assay reproducibility	Throughput and clinical integration	Lu et al. (2026)
Vascularized platforms	Perfusable constructs with enhanced transport and signaling	Support channel formation and multicellular patterning	Optimize architecture-perfusion-function relationships	Perfusability, barrier integrity, endothelial organization, tissue maturation, perfusion stability	System complexity and scalability	Kim and Cho (2024), Yin et al. (2024)
Multifunctional/organ-on-chip platforms	Integrated systems with sensing, conductivity, or adaptive function	Provide structural support plus higher-order functionality	Support multimodal optimization and evaluation	Functional integration, platform stability, tissue complexity, perturbation response, multimodal readouts	Standardization and multimodal data integration	Konwarh (2025), Papamichail et al. (2025)

Abbreviations: AI, artificial intelligence; ECM, extracellular matrix.

Several emerging tissue targets illustrate this trend. In soft tissue regeneration, self-healing hydrogel bioinks are promising because they combine printability with post-printing durability and biomimetic support for skin, cartilage, nerve, and cardiac tissue (Pourmokhtari et al., 2025; Dai et al., 2025). In neural tissue engineering, functional biomaterials and 3D bioprinting are being linked to more precise scaffold architectures for peripheral nerve repair and spinal cord-related applications (Li S. et al., 2025). In regenerative dentistry, AI-driven bioprinting may support more precise and personalized regeneration of teeth, periodontal tissues, and alveolar bone (Liang et al., 2025). Another important direction is *in situ* regenerative bioprinting, which deposits reactive bioinks directly at the defect site. This approach may improve tissue integration and clinical relevance, but it also places higher demands on bioink responsiveness, printing precision, and environmental adaptability (Huang et al., 2025). For smart hydrogels, AI may help match bioink composition and process settings to variable defect geometries and local tissue conditions (Zhang et al., 2025b; Dhiman et al., 2026). Recent regenerative medicine reviews increasingly describe *in situ* bioprinting as a future-oriented area in which bioink intelligence and process intelligence need to converge (Huang et al., 2025; Zhang Z. et al., 2024).

Regenerative tissue engineering therefore illustrates how AI can connect formulation, process, structure, and function rather than only improve fabrication efficiency (Jiang et al., 2025; Zhang Z. et al., 2024). The most useful models will be those that link bioink chemistry, crosslinking, rheology, printing settings, and construct architecture to tissue-specific maturation and function (Theus et al., 2020; Kim, 2023; Son et al., 2025). Across applications, AI-assisted workflows should be evaluated by whether they make the formulation–process–structure–function chain more measurable and reproducible (Grijalva Garces et al., 2023; Jiang et al., 2025). Validation endpoints should remain application-specific: vascular constructs require perfusion and barrier function; neural constructs require network activity and electrophysiology; cardiac constructs require synchronized contraction; and bone, cartilage, and musculoskeletal targets require matrix deposition, mineralization when relevant, mechanical maturation, and load-bearing behavior (Li S. et al., 2025; Zhang et al., 2026; Galarraga et al., 2019; Jaouher et al., 2025; Theus et al., 2020). This framing preserves the need for long-term maturation and tissue-specific function and prevents a narrow manufacturing endpoint from being presented as evidence of tissue-level success (Kim, 2023; Son et al., 2025).

### 6.2 *In vitro* disease models

*In vitro* disease models link biofabrication with mechanistic research and preclinical evaluation. Unlike regenerative applications, disease models aim to reproduce pathophysiological microenvironments in controlled formats. 3D bioprinting can spatially organize multiple cell types within hydrogel matrices, better recapitulating tissue architecture and heterogeneity than conventional 2D culture (Tao et al., 2025; Hua and Gaharwar, 2026; Wiewiórska-Krata et al., 2026). Tumor models are currently the most developed: 3D bioprinting can reconstruct multicellular organization, stromal interactions, and extracellular matrix context, while AI supports model optimization, image analysis, and phenotype interpretation. This combination is attractive because tumor modeling requires integration of multiple variables difficult to optimize empirically (Kurzątkowska et al., 2025; Piotrowska et al., 2025; Weng et al., 2025). Disease modeling extends beyond oncology to autoimmune and inflammatory diseases including rheumatoid arthritis, type 1 diabetes, inflammatory bowel disease, systemic lupus erythematosus, and neuroinflammatory conditions (Hua and Gaharwar, 2026; Wiewiórska-Krata et al., 2026). AI is especially valuable because disease models are inherently multidimensional; their relevance depends on spatial cell organization, matrix patterns, emergent phenotypes, and dynamic responses to perturbation. AI can identify formulation-outcome relationships and extract quantitative descriptors from complex imaging data (Weng et al., 2025; Dhiman et al., 2026). Bioprinted models are also emerging as new approach methodologies for drug testing, because they better reproduce native tissue architecture than traditional 2D systems, broadening the value of AI-assisted bioprinting from printing structures to generating models for mechanistic studies and therapeutic evaluation (Hua and Gaharwar, 2026).

For disease modeling, the central AI task is to improve the reproducibility and interpretability of biological phenotypes rather than to maximize printability alone. Useful models should relate formulation and process variables to drug response, invasion, hypoxia, tumor-stromal interaction, immune or vascular microenvironmental features, disease-specific marker expression, and long-term phenotype stability. These endpoints allow bioprinted disease models to serve as more reliable experimental systems instead of visually consistent but biologically under-validated constructs.

### 6.3 Drug screening and personalized testing

Drug screening and personalized testing are among the most translationally important application scenarios for AI-assisted smart hydrogel bioprinting. Beyond generating structurally complex tissues, bioprinting can create experimentally accessible platforms that better reproduce human-relevant cellular organization, extracellular matrix context, and treatment response than conventional 2D culture. Recent reviews increasingly describe AI-augmented bioprinting as a data-driven bridge between biofabrication and pharmacological research (Budharaju et al., 2025; Yang K. et al., 2024; Wang et al., 2025). A major advantage of these platforms is that they improve physiological relevance while retaining experimental controllability. This is especially important for smart hydrogel bioinks, whose responsive and tunable properties can better mimic tissue-specific stiffness, transport behavior, and cell–matrix interactions, thereby improving the interpretability of treatment responses (Budharaju et al., 2025). This translational value is particularly evident in oncology. Recent reviews describe 3D bioprinted cancer models as promising platforms for drug discovery and development because they can better reproduce tumor heterogeneity and improve the predictive value of drug screening compared with conventional 2D *in vitro* systems (Kurzątkowska et al., 2025). When combined with patient-derived cells or tumor-relevant bioinks, these models may further support more personalized testing workflows.

AI further strengthens this application space by linking complex construct features with treatment-related outcomes. Rather than relying only on endpoint viability assays or visual inspection, AI can support multiparametric analysis of imaging, morphology, and phenotypic data, and identify patterns associated with drug susceptibility or resistance. A representative glioma study combined 3D bioprinting with machine learning to identify susceptibility patterns and microenvironment characteristics, illustrating how AI can extract therapeutically relevant information from complex printed tumor systems (Wang et al., 2025). The role of bioprinting becomes even more compelling in personalized testing. A recent study on intrahepatic cholangiocarcinoma reported patient-derived 3D-bioprinted models that recapitulated tumor autologous traits and were used to predict personalized adjuvant therapy, highlighting the potential of bioprinted systems for individualized functional testing. More broadly, patient-derived 3D-bioprinted models may help bridge the gap between conventional preclinical testing and precision medicine by providing faster and more biologically relevant platforms for drug sensitivity assessment (Lu et al., 2026). The value of AI in this setting is therefore strongest when it integrates design and evaluation: selecting bioinks compatible with a target tissue, predicting process windows, quantifying construct architecture, and linking early readouts to later tissue-specific function. This application-oriented synthesis avoids treating all printed constructs as equivalent and helps define which evidence is sufficient for a regenerative, disease-modeling, or personalized-testing claim. The relative weight of these endpoints should be defined before model development. A vascular construct may prioritize perfusability and barrier function, whereas a tumor model may prioritize drug response, invasion, and hypoxia. This avoids training models on convenient measurements while making claims about biological functions that were not measured.

### 6.4 Vascularized/multifunctional platforms

Vascularized and multifunctional bioprinted platforms represent an advanced application of AI-assisted smart hydrogel bioprinting because they move beyond single-function constructs toward systems with greater transport, signaling, and tissue-level complexity. In particular, vascularized platforms seek to create perfusable architectures that support nutrient and oxygen delivery, waste removal, and biologically meaningful cell–cell communication. Because vascularization remains a major barrier to graft survival, construct maturation, and clinical translation, it has become an important test case for intelligent biofabrication strategies (Kim and Cho, 2024; Yin et al., 2024; Son et al., 2025). Smart hydrogel bioprinting is especially valuable in this setting because it combines tunable material behavior with spatial control over channel architecture and multicellular organization. Recent work on vascularized constructs and organ-on-a-chip systems highlights growing interest in perfusable hydrogel-based platforms that integrate endothelial structures with tissue-specific compartments, suggesting that vascularization is increasingly being treated as a scalable platform capability rather than an isolated feature (Fritschen et al., 2024; de Barros et al., 2025; Jäger et al., 2026).

AI adds value by helping manage the greater design complexity of these systems. Compared with simpler constructs, vascularized platforms require coordinated optimization of channel geometry, bioink properties, perfusion compatibility, multicellular patterning, and functional readouts. AI-assisted methods are therefore well suited to identifying formulation–architecture–function relationships, improving design efficiency, and supporting richer evaluation of perfusable constructs (Silva Robazzi et al., 2025; Dhiman et al., 2026). The notion of multifunctionality extends this concept further. Hydrogel-based platforms are increasingly being engineered not only for structural support, but also for additional biomedical functions such as self-healing behavior, adaptive responses, and implantable integration (Nagay et al., 2026). Conductive and interfacial hydrogel systems also provide opportunities for sensing and bioelectronic integration, particularly in flexible electronic applications (Song et al., 2026). In regenerative bioprinting, these trends suggest that future platforms may combine tissue formation, monitoring, and responsive actuation rather than serving only as passive scaffolds (Zhang et al., 2026).

This direction is also relevant to organoid and organ-on-chip applications. Bioprinted organoids can improve architectural control and spatial organization, increasing their value for disease modeling, drug testing, and biomedical research (Li Z. et al., 2025). Organoids-on-chip further enhance tissue function by using microfluidic systems to create more physiologically relevant culture environments (Papamichail et al., 2025). Vascularized organoid-on-chip platforms extend this approach by incorporating perfusable vascular features, which can improve microenvironmental control and translational relevance (Konwarh, 2025). Scalable and reproducible organoid production is also essential for broader application and manufacturing-oriented translation (Kim et al., 2026). Personalized and precision medicine applications raise especially strict validation requirements because patient-derived cells, donor variability, and patient-specific phenotypes can strongly shift model behavior. AI-assisted workflows may help compare donor-specific bioink responses, printing robustness, maturation trajectories, and drug sensitivity, but these predictions should be evaluated with leave-one-donor-out or external validation when the intended use involves patient-specific deployment.

## 7 Current bottlenecks and unresolved challenges

### 7.1 Lack of standardized datasets and reporting criteria

A fundamental barrier to AI-assisted smart hydrogel bioprinting is the lack of standardized datasets and reporting criteria. Although the field is generating increasing amounts of formulation, rheological, process, imaging, and biological data, these data are often reported in fragmented and non-comparable formats. Similar materials may be described using different naming conventions, key rheological descriptors may be incompletely reported, and critical printing variables such as nozzle size, pressure, temperature, or crosslinking conditions are not always documented in a consistent manner. As a result, datasets collected from different studies frequently differ not only in structure, but also in meaning and interpretability (Tănase et al., 2025; Maddalon et al., 2026). This problem is especially important for AI because predictive models require sufficiently consistent inputs and outputs across experiments. In bioprinting, however, even commonly used endpoints such as printability are not yet universally defined. Recent work has emphasized the need for a consolidated definition of bioink printability in additive manufacturing, as the term is still used inconsistently across studies and application contexts (Resende et al., 2025). The lack of harmonized reporting also directly limits reproducibility and cross-study comparison. A recent Joint Research Centre report on 3D bioprinting standards identified reproducibility, scalability, and bioink standardization as persistent challenges. It also emphasized the role of standardization in improving quality and safety (Maddalon et al., 2026). This concern is consistent with recent evidence-mapping work in biomedical 3D printing, which shows that AI-related studies are increasing rapidly but remain distributed across diverse and only partly connected research domains (Tănase et al., 2025). Under these conditions, it is difficult to determine whether differences in model performance reflect true methodological progress or differences in local experimental practice, label definitions, or reporting depth.

Poor standardization also complicates multimodal data integration. Smart hydrogel bioprinting often requires material descriptors, rheological measurements, process parameters, imaging outputs, and biological readouts to be linked within a unified learning framework. When these data are generated using different conventions, time points, and quality criteria, they become sparse, noisy, and difficult to align computationally. This fragmentation weakens model generalizability and limits the development of AI systems that can transfer across bioinks, printers, and applications. The implications extend beyond model training. Insufficient standardization also affects quality assurance, regulatory interpretation, and the translation of bioprinted products (Maddalon et al., 2026). Standardized datasets and reporting criteria are therefore not only technical requirements for AI development, but also essential foundations for reliable and clinically credible biofabrication. Future progress will require clearer endpoint definitions, more complete reporting of material and process variables, and interoperable datasets that connect formulation, printing, characterization, and functional outcomes. Without these foundations, even advanced AI models will remain difficult to compare, validate, and generalize.

### 7.2 Limited model generalizability across materials, printers, and biological systems

Another bottleneck in AI-assisted smart hydrogel bioprinting is the limited generalizability of current models across materials, printing platforms, and biological systems. Many machine learning frameworks perform well only within the experimental settings in which they were trained, but their performance often declines when applied to different bioinks, printer configurations, imaging setups, or cellular contexts. Because construct quality and biological outcomes depend on coupled interactions among formulation chemistry, rheology, hardware, and living-cell responses, models often capture local correlations rather than broadly transferable rules (Mohammadnabi et al., 2025).

This limitation arises at multiple levels. Hydrogel systems differ in molecular architecture, crosslinking mechanisms, rheological behavior, and bioactivity, so the same features may not have the same predictive meaning across formulations. Printer mechanics, nozzle geometry, extrusion modes, and software pipelines further alter fabrication behavior. Biological variables such as cell type, density, culture duration, and endpoint selection add another layer of variability. Recent evidence-mapping work suggests that biomedical 3D printing remains distributed across only partially connected research themes, helping explain why locally successful models do not readily translate into broadly usable systems (Tănase et al., 2025). The challenge is also reflected in recent attempts to improve portability. A 2025 study showed that transfer-learning strategies can adapt models across experimental settings while reducing the need for extensive retraining, indicating that poor portability is already a practical concern in this field (Bracco et al., 2025). Recent reviews further emphasize that robust deployment will require models that can tolerate pre-process, in-process, and post-process variability rather than operating only within isolated experimental silos. Better standardization, larger and more diverse datasets, and transfer-learning or domain-adaptation strategies may all help improve robustness and reuse across printing contexts (Mohammadnabi et al., 2025; Salas et al., 2026).

Limited generalizability across materials, printers, and biological systems therefore remains a major unresolved challenge for AI-assisted smart hydrogel bioprinting. Until models can better tolerate variation in formulation space, hardware environment, and biological context, their practical value will remain constrained by local calibration and narrow experimental boundaries.

### 7.3 Difficulties in multimodal data integration and feature representation

A further challenge in AI-assisted smart hydrogel bioprinting is multimodal data integration and feature representation. Relevant information spans formulation composition, rheology, printing parameters, imaging outputs, and biological outcomes, but these data are generated at different scales and in different formats. As a result, material–process–structure–function relationships are difficult to encode in a unified and machine-readable form (Ramesh et al., 2024; Wang et al., 2025). This problem is compounded by context dependence. The predictive meaning of material descriptors such as viscosity or yield stress can change with nozzle geometry, extrusion mode, temperature, and cell system, while image-derived morphology and biological readouts add further heterogeneity in format, timing, and interpretation. Current feature representations are therefore often too shallow or too study specific to support robust transferability and biological interpretation (Sun et al., 2023; Rafieyan et al., 2024). Progress will require better representation of coupled formulation–process–structure–function relationships, rather than simply adding more variables to larger datasets. Standardized analytical pipelines and interoperable descriptors are needed to improve the quantification and comparability of bioink performance (Strauß et al., 2023). Data structures that preserve experimental context will also be important for building AI models that are more interpretable, comparable, and generalizable, particularly when bioprinting data are integrated with multi-algorithm machine learning approaches (Tang et al., 2024).

### 7.4 Bridging short-term printing performance and long-term tissue functionality

Most current AI models in hydrogel bioprinting still emphasize short-term and manufacturing-centered endpoints, including rheology, filament morphology, geometric fidelity, printing resolution, positional error, and immediate cell viability (Zhang C. et al., 2024; Sarah et al., 2025; Wang et al., 2025). These endpoints are important because they determine whether a construct can be fabricated reproducibly (Hölzl et al., 2016; Schwab et al., 2020; Theus et al., 2020). However, fewer models evaluate long-term proliferation, differentiation, extracellular matrix deposition, tissue maturation, or tissue-specific function. This imbalance limits the ability of AI-assisted design to predict whether a printable construct will become a functional tissue model or regenerative construct (Kim, 2023; Wang et al., 2025).

Printing performance and tissue functionality can also become decoupled. High crosslinking density may improve shape fidelity while restricting cell migration, nutrient transport, and matrix remodeling (Schwab et al., 2020; Kim, 2023). High immediate viability does not guarantee long-term phenotype stability, differentiation, or maturation (Theus et al., 2020; Kim, 2023). Geometric deviation may indicate printing failure, but it may also reflect cell-mediated contraction or beneficial tissue remodeling. Excellent printability therefore does not necessarily translate into successful tissue development (Schwab et al., 2020; Theus et al., 2020; Kim, 2023). Future models should integrate manufacturing endpoints, structural endpoints, cellular endpoints, and functional endpoints in time-resolved and multi-objective frameworks rather than compressing complex outcomes into a single printability score (Wang et al., 2025). Realistic progress is limited by scarce longitudinal datasets, inconsistent endpoint definitions, high costs of repeated biological assays, missing data across time points, donor and batch effects, limited external validation, and the difficulty of linking early predictors to late function.

### 7.5 Model validation, grouped data splitting, and leakage prevention

Model validation is a central challenge because observations in bioink datasets are often not independent samples (Little et al., 2017; Bradshaw et al., 2023). Multiple rheological measurements may come from the same formulation; multiple specimens may come from the same batch; multiple filaments may come from the same printing run; multiple image fields may come from the same construct; multiple constructs may share a donor or cell preparation; video frames may belong to the same sequence; augmented images may derive from the same original image; and technical replicates may share the same experimental condition. Random row-level splitting can therefore place highly related observations into both training and test sets, producing overly optimistic performance estimates (Rouzrokh et al., 2022; Tampu et al., 2022; Kapoor and Narayanan, 2023).

Grouped validation should match the level at which the model is expected to generalize (Saeb et al., 2017; Little et al., 2017; Bradshaw et al., 2023). Random row-level splitting can produce overly optimistic estimates when samples are correlated within formulations, batches, printing runs, constructs, donors, image fields, or laboratories. Leave-one-formulation-out validation tests generalization to unseen formulations; leave-one-batch-out validation tests batch-to-batch robustness; leave-one-printing-run-out validation prevents leakage among samples from the same run; leave-one-construct-out validation avoids leakage among repeated images, slices, fields, or measurements from the same construct; leave-one-donor-out validation is needed for donor-derived or patient-specific systems; leave-one-printer-out and leave-one-laboratory-out validation test hardware transferability and external deployment. Nested cross-validation should be used for model selection and hyperparameter tuning in small datasets, while external validation remains the strongest test of cross-platform and cross-laboratory generalization (Cawley and Talbot, 2010). Data leakage must be treated as a central risk rather than a minor technical detail. Benchmarking should also be matched to the task. Regression models for viscosity, modulus, strand width, or viability should report mean absolute error, root mean square error, coefficient of determination, calibration error, and prediction intervals where possible. Classification models for printability or defect labels should report accuracy together with balanced accuracy, F1-score, Matthews correlation coefficient, area under the receiver operating characteristic curve, and area under the precision-recall curve when classes are imbalanced. Segmentation models should report Dice coefficient, intersection over union, and pixel-level precision and recall, preferably with construct-level grouping. Optimization studies should report not only the best objective achieved, but also the number of experiments saved, robustness of the process window, and prospective confirmation of selected candidates.

### 7.6 Uncertainty estimation and out-of-distribution detection

Uncertainty estimation is needed because model predictions in bioink development may influence formulation choice, process windows, construct quality, and biological interpretation. Aleatoric uncertainty arises from measurement noise, batch variability, biological heterogeneity, cell-to-cell variability, and stochastic printing behavior; adding more data may not fully eliminate it (Kendall and Gal, 2017). Epistemic uncertainty arises from limited model knowledge or incomplete coverage of the training domain, such as small datasets, sparsely sampled formulation spaces, limited material diversity, and unobserved printers or biological systems; representative additional data can often reduce it (Kendall and Gal, 2017; He et al., 2026). Candidate methodological options include Gaussian processes, model ensembles, Monte Carlo dropout, quantile regression, and conformal prediction, although systematic comparison in smart hydrogel bioink datasets remains limited (Gal and Ghahramani, 2016; Lakshminarayanan et al., 2017).

Out-of-distribution (OOD) detection is equally important. Smart hydrogel bioink models may encounter a new polymer chemistry, crosslinking mechanism, concentration range, molecular weight, degree of substitution, nozzle diameter, extrusion pressure range, printer hardware, support bath, temperature condition, light-curing condition, cell type, donor, cell density, imaging modality, laboratory-specific imaging setting, annotation protocol, culture duration, or maturation condition. In these settings, uncertainty-aware systems should flag unsafe extrapolation, identify predictions that should not be trusted, prioritize additional experiments, select candidates for confirmatory validation, decide whether a formulation is safe to print or biologically test, trigger human review, and support safer transition from printability prediction to biological validation (Hendrycks and Gimpel, 2017; Yang J. et al., 2024; Lookman et al., 2019).

Uncertainty should guide experimental decisions rather than serve only as a model diagnostic. Gaussian processes, ensembles, Monte Carlo dropout, quantile regression, conformal prediction, calibration analysis, and descriptor- or representation-space distance can help identify predictions that require additional validation. Table 5 summarizes practical validation designs, task-specific metrics, and OOD triggers. In practice, high-uncertainty formulations may help expand the model domain but should not be treated as validated candidates without confirmatory experiments. Low-uncertainty predictions can support routine screening only when the endpoint, split strategy, and calibration are clearly reported.

**TABLE 5:** Practical validation, benchmarking, and OOD-risk checklist for AI-assisted smart hydrogel bioink workflows.

Validation or risk-control item	Generalization question or decision use	Recommended metrics or actions
Leave-one-formulation-out validation	Can the model generalize to unseen formulations?	Report regression or classification performance on held-out formulations; avoid mixing formulation variants across train/test sets
Leave-one-batch-out validation	Is performance robust to batch-to-batch variation?	Hold out full preparation batches; report calibration error and performance variability across batches
Leave-one-printing-run-out validation	Does performance survive run-level correlation?	Hold out complete printing runs to prevent leakage among highly correlated samples
Leave-one-construct-out validation	Can the model generalize beyond repeated images, slices, fields, or measurements from the same construct?	Group images and repeated measures by construct; report field-level and construct-level metrics separately when possible
Leave-one-donor-out validation	Can donor-derived or patient-specific systems generalize to unseen donors?	Hold out all samples from a donor; report donor-level robustness and failure modes
Leave-one-printer or leave-one-laboratory-out validation	Can the model transfer across hardware or external sites?	Hold out one printer or laboratory; use external validation for deployment claims
Nested cross-validation	Is model selection unbiased in small datasets?	Use inner loops for tuning and outer loops for performance estimation
Regression tasks	How accurate and calibrated are continuous predictions?	Mean absolute error (MAE), root mean square error (RMSE), R2, calibration error, prediction intervals
Classification tasks	How reliable are categorical printability or quality labels?	Accuracy, balanced accuracy, F1-score, Matthews correlation coefficient (MCC), area under the receiver operating characteristic curve (AUROC), area under the precision-recall curve (AUPRC)
Segmentation tasks	How accurate are image-derived structure measurements?	Dice coefficient, intersection over union (IoU), pixel-level precision/recall, grouped image-field validation
Optimization tasks	Does the strategy reduce experimental burden while improving outcomes?	Number of experiments saved, best achieved objective, robustness of process window, prospective confirmation
Biological prediction tasks	Do early descriptors predict late biological performance?	Beyond short-term viability: proliferation, apoptosis, phenotype maintenance, differentiation, ECM deposition, maturation, tissue-specific function, drug response, and long-term stability
Smart hydrogel bioink OOD triggers	When should predictions be flagged or reviewed?	New chemistry, crosslinking, concentration, molecular weight, degree of substitution, nozzle, pressure, hardware, support bath, temperature, light curing, cell type, donor, cell density, imaging modality, annotation protocol, culture duration, or maturation condition

Abbreviations: OOD, out-of-distribution; MAE, mean absolute error; RMSE, root mean square error; R^2^, coefficient of determination; MCC, Matthews correlation coefficient; AUROC, area under the receiver operating characteristic curve; AUPRC, area under the precision-recall curve; IoU, intersection over union.

### 7.7 Interpretability, regulatory translation, and evidence levels

Interpretability and evidence grading remain enabling conditions for credible deployment. Direct smart hydrogel bioink evidence should be distinguished from broader bioink or extrusion-bioprinting evidence, adjacent-field methodological inspiration from materials science, drug discovery, robotic chemistry, or general hydrogel informatics, and conceptual or prospective applications. This distinction is especially important for foundation models, AI agents, and autonomous laboratories, where much of the current evidence comes from adjacent fields. Regulatory translation adds another layer of complexity because bioprinted constructs may overlap with medical devices, biologics, tissue-engineered products, or combination products, and AI-assisted decisions may require traceable documentation, fit-for-purpose validation, lifecycle oversight, and evidence linking model outputs to quality and safety (Perin et al., 2026; Lekadir et al., 2025; U.S. Food and Drug Administration, 2025; U.S. Food and Drug Administration, 2026).

Evidence grading should match the intended claim. Models validated within one printer, material family, donor source, or imaging protocol can support local use, but they cannot justify cross-platform, patient-specific, or multicenter claims without external validation. Translational relevance therefore requires traceable datasets, predefined endpoints, uncertainty calibration, grouped validation, and clear separation between decision support and automated action.

## 8 Discussion

Smart hydrogel bioinks are important platforms for 3D bioprinting because they combine hydrated, cell-supportive environments with tunable and dynamic functions. In this context, the main value of AI-assisted methods is not only faster screening, but also a clearer workflow logic linking formulation design, feature representation, property prediction, process optimization, monitoring, construct validation, and evidence maturity. This review therefore separates prediction, optimization, monitoring, feedback control, and prospective autonomous workflows rather than treating them as equivalent forms of automation.

This distinction helps avoid over-extrapolation. Current evidence in smart hydrogel bioinks mainly supports selected prediction, printability assessment, offline optimization, and image-based characterization tasks. Broader bioprinting studies provide useful evidence for monitoring and isolated feedback correction. By contrast, foundation models, transfer learning from non-hydrogel domains, AI agents, robotic chemistry, and self-driving laboratories remain adjacent-field inspirations or prospective concepts unless validated in hydrogel-specific formulation, printing, culture, and functional endpoint settings.

Another key implication is that fabrication-centered endpoints are insufficient. Printability, geometric fidelity, and immediate cell viability are necessary early gates, but they do not establish tissue engineering success. Future AI-assisted bioink design should connect early material and process descriptors with long-term biological function, including maturation, matrix remodeling, tissue-specific behavior, disease phenotype, drug response, and stability across batches, donors, printers, and laboratories. AI-assisted smart hydrogel bioprinting is therefore best viewed as an evidence-stratified workflow under development. Progress will depend on standardized reporting, shared datasets, grouped and external validation, uncertainty-aware decision rules, OOD detection, function-oriented endpoints, and human oversight. These requirements do not limit the potential of autonomous workflows; they define the evidence needed before such workflows can be considered reliable for bioink discovery and translational tissue engineering.
